# Reconstructing SALMFamide Neuropeptide Precursor Evolution in the Phylum Echinodermata: Ophiuroid and Crinoid Sequence Data Provide New Insights

**DOI:** 10.3389/fendo.2015.00002

**Published:** 2015-02-02

**Authors:** Maurice R. Elphick, Dean C. Semmens, Liisa M. Blowes, Judith Levine, Christopher J. Lowe, Maria I. Arnone, Melody S. Clark

**Affiliations:** ^1^School of Biological and Chemical Sciences, Queen Mary University of London, London, UK; ^2^Hopkins Marine Station, Stanford University, Pacific Grove, CA, USA; ^3^Stazione Zoologica Anton Dohrn, Naples, Italy; ^4^British Antarctic Survey, Cambridge, UK

**Keywords:** neuropeptide, echinoderm, SALMFamide, evolution, ophiuroid, crinoid

## Abstract

The SALMFamides are a family of neuropeptides that act as muscle relaxants in echinoderms. Analysis of genome/transcriptome sequence data from the sea urchin *Strongylocentrotus purpuratus* (Echinoidea), the sea cucumber *Apostichopus japonicus* (Holothuroidea), and the starfish *Patiria miniata* (Asteroidea) reveals that in each species there are two types of SALMFamide precursor: an L-type precursor comprising peptides with a C-terminal LxFamide-type motif and an F-type precursor solely or largely comprising peptides with a C-terminal FxFamide-type motif. Here, we have identified transcripts encoding SALMFamide precursors in the brittle star *Ophionotus victoriae* (Ophiuroidea) and the feather star *Antedon mediterranea* (Crinoidea). We have also identified SALMFamide precursors in other species belonging to each of the five echinoderm classes. As in *S. purpuratus*, *A. japonicus*, and *P. miniata*, in *O. victoriae* there is one L-type precursor and one F-type precursor. However, in *A. mediterranea* only a single SALMFamide precursor was found, comprising two peptides with a LxFamide-type motif, one with a FxFamide-type motif, five with a FxLamide-type motif, and four with a LxLamide-type motif. As crinoids are basal to the Echinozoa (Holothuroidea + Echinoidea) and Asterozoa (Asteroidea + Ophiuroidea) in echinoderm phylogeny, one model of SALMFamide precursor evolution would be that ancestrally there was a single SALMFamide gene encoding a variety of SALMFamides (as in crinoids), which duplicated in a common ancestor of the Echinozoa and Asterozoa and then specialized to encode L-type SALMFamides or F-type SALMFamides. Alternatively, a second SALMFamide precursor may remain to be discovered or may have been lost in crinoids. Further insights will be obtained if SALMFamide receptors are identified, which would provide a molecular basis for experimental analysis of the functional significance of the “cocktails” of SALMFamides that exist in echinoderms.

## Introduction

The SALMFamides are a family of neuropeptides that occur in species belonging to the phylum Echinodermata (e.g., starfish, sea cucumbers, and sea urchins) (1). The prototypes for this neuropeptide family were discovered in the starfish species *Asterias rubens* and *Asterias forbesi* and are known as SALMFamide-1 (S1) and SALMFamide-2 (S2) (2, 3). S1 was identified as a C-terminally amidated octapeptide with the amino acid sequence Gly-Phe-Asn-Ser-Ala-Leu-Met-Phe-NH_2_ (GFNSALMFamide) and S2 was identified as a C-terminally amidated dodecapeptide with the amino acid sequence Ser-Gly-Pro-Tyr-Ser-Phe-Asn-Ser-Gly-Leu-Thr-Phe-NH_2_ (SGPYSFNSGLTFamide). Both peptides have the C-terminal motif FNSxLxFamide (where *x* is variable), which suggested that S1 and S2 may have evolved as a consequence of gene duplication or intragenic DNA duplication. Immunocytochemical investigation of the expression of S1 and S2 in *A. rubens* revealed widely distributed patterns of expression in the nervous system but in separate populations of neurons (4, 5). Furthermore, S1 and S2 are present in the innervation of neuromuscular organs and, consistent with this finding, both peptides cause relaxation of starfish cardiac stomach, tube foot, and apical muscle preparations *in vitro* (6–9).

Subsequent to the discovery of S1 and S2 in starfish, SALMFamide-type neuropeptides were identified in other echinoderms. Thus, SALMFamide-type peptides were isolated from two sea cucumber species – *Holothuria glaberrima* and *Apostichopus japonicus* – and, consistent with the actions of S1 and S2 in starfish, these peptides cause muscle relaxation in sea cucumbers (10–12). It appears, therefore, that the relaxing action of SALMFamides on echinoderm muscle may be a general property of this neuropeptide family (9). Discovery of SALMFamides in sea cucumbers also revealed structural heterogeneity in SALMFamides. Two SALMFamides isolated from *Holothuria glaberrima* were identified as GFSKLYFamide and SGYSVLYFamide, which share with the starfish SALMFamides S1 and S2 the C-terminal motif SxLxFamide (i.e., L-type SALMFamides) (11). However, two SALMFamides isolated from *Apostichopus japonicus* were identified as GYSPFMFamide and FKSPFMFamide, which have the C-terminal motif SxFxFamide (i.e., F-type SALMFamides) (12).

Sequencing of the genome and transcriptome of the sea urchin *Strongylocentrotus purpuratus* (class Echinoidea) provided the first insight into the genetic basis of SALMFamide-type neuropeptide diversity in an echinoderm species (13). Thus, in *S. purpuratus* there are two genes encoding SALMFamide precursor proteins: one gene encodes a precursor comprising one L-type SALMFamide and one L-type-like (SxIxFamide) SALMFamide (14) and the second gene encodes a precursor comprising seven F-type SALMFamides (15). However, a more complicated picture has emerged as sequence data from other echinoderms has become available. The Holothuroidea (sea cucumbers) are a sister group to the class Echinoidea and, as in *S. purpuratus*, analysis of transcriptome sequence data from *A. japonicus* revealed two SALMFamide precursor transcripts. One of the precursors comprises three L-type or L-type-like SALMFamides and is homologous to the L-type SALMFamide precursor in *S. purpuratus* that contains two L-type or L-type-like SALMFamides (16). The second SALMFamide precursor in *A. japonicus* is largely comprised of F-type or F-type-like SALMFamides (five in total) but interestingly, unlike the precursor that gives rise to F-type SALMFamides in *S. purpuratus*, it also contains three L-type/L-type-like SALMFamides (16, 17). Thus, the “cocktail” of SALMFamides in *A. japonicus* is more complex than in *S. purpuratus*. What is not clear from these data, however, is which condition is ancestral and which is derived and to address this issue sequence data from other echinoderms is required.

Recently, genome sequence data for the starfish *Patiria miniata* has been obtained and this has revealed a SALMFamide profile similar to the sea cucumber *A. japonicus*. Thus, in *P. miniata* one SALMFamide precursor is solely comprised of L-type SALMFamides, which include S1 and six other structurally related peptides (16). The other SALMFamide precursor in *P. miniata* is largely comprised of F-type or F-type-like SALMFamides (eight in total) but it also contains an S2-like peptide with an L-type C-terminal motif (16). These predicted *P. miniata* SALMFamide precursor sequences now require confirmation by transcript sequencing. Nevertheless, the data currently available suggest that the occurrence of a precursor comprising several F-type SALMFamides and one or more L-type SALMFamides may be the ancestral condition, with the absence of L-type SALMFamides in the sea urchin F-type SALMFamide precursor being a derived condition. However, there remains the possibility that the occurrence of L-type SALMFamides in the F-type SALMFamide precursor is a feature that has arisen independently in both the holothurian and asteroid lineages. To gain further insight on this issue it will be necessary to determine the sequences of SALMFamide precursors in species belonging to two other extant echinoderm classes: the Ophiuroidea (brittle stars) and the Crinoidea (feather stars and sea lilies). As a sister group to the Asteroidea (starfish), the Ophiuroidea could provide key insights on SALMFamide precursor evolution. Thus, if the F-type SALMFamide precursor in brittle stars also contains L-type or L-type like SALMFamides, as in starfish and sea cucumbers, this would add weight to the notion that this is a feature that dates back to the common ancestor of the Asterozoa (Asteroidea + Ophiuroidea) and the Echinozoa (Holothuroidea + Echinoidea). The Crinoidea are basal to the Asterozoa and the Echinozoa (18) and determination of the sequences of SALMFamide precursors in species belonging to this class of echinoderms could provide insight into the ancestral condition in the common ancestor of all extant echinoderms.

Here, we have analysed transcriptome sequence data from the starfish *P. miniata*, which in combination with genome sequence data has enabled definitive determination of the sequences of SALMFamide precursors in this species. Importantly, these data have also enabled comparison of SALMFamide gene structure in an asterozoan species (*P. miniata*) and an echinozoan species (*S. purpuratus*). Furthermore, here we report the sequences of novel SALMFamide precursors that we have discovered by analysis of transcriptome sequence data from the ophiuroid *Ophionotus victoriae*, a brittle star species that has a circumpolar distribution around Antarctica, and the crinoid *Antedon mediterranea*, a feather star species that is (as its name implies) native to the Mediterranean Sea. Having identified SALMFamide precursors in single species from each of the five echinoderm classes, we investigated the generality of our findings by analysis of genome/transcriptome sequence data from other echinoderm species.

## Materials and Methods

### Transcriptome sequencing of the starfish *Patiria miniata*

*Patiria miniata* transcriptome sequence was produced using RNA from several developmental stages from blastula to juveniles, including bipinnaria and brachiolaria larvae. Sequencing was carried out at Huntman Genome Center University of Utah using Illumina HiSeq 101 paired end sequencing. 172,091,442 paired end reads were lightly trimmed and adapter sequences removed using Trimmomatic (19) with the following parameters: ILLUMINACLIP:illuminaClipping.fa:2:40:15 LEADING:3 TRAILING:3 SLIDINGWINDOW:4:20 MINLEN:25. Post-quality trimming was assessed using FastQC^1^. High-quality, trimmed reads were assembled *de novo* using the Trinity suite of programs, with default parameters except min_kmer_cov = 2 (20). The assembly yielded 203,888 transcripts, representing 101,664 transcript groups (genes). The contig N50 was 1717 bp, the median contig length was 502 bp, and the total assembled bases were 194,282,131. If only the longest isoform in each transcript group was considered, then the contig N50 dropped to 1490 bp, the median contig length was 389 bp, and the total assembled bases were 81,494,371.

### Transcriptome sequencing of the brittle star *Ophionotus victoriae* and the feather star *Antedon mediterranea*

Arms dissected from a single adult specimen of *O. victoriae* and arms dissected from a single adult specimen of *A. mediterranea* were used for RNA isolation (Total RNA Isolation System, Promega, Southampton, UK). Library preparation (TruSeqv2 kit, Illumina, Little Chesterford, Essex, UK) was performed at the QMUL Genome Center and sequencing (Illumina HiSeq 2500 platform) was performed at the BRC Genomics Core Facility at Guy’s and St Thomas’ NHS Foundation Trust and King’s College London.

Illumina HiSeq sequencing yielded 155931609 and 116089417 paired 101 bp long reads for *O. victoriae* and *A. mediterranea*, respectively. Raw sequence data were assembled using the de Brujn graph assembler Short Oligonucleotide Analysis Package SOAPdenovo-Trans-31mer 1.03^2^ with the Kmer value set to 31, employing use of a high performance computing system (Apocrita^3^). The *O. victoriae* assembly yielded 669,744 contigs, with a mean length of 243 bp and N50 of 297 bp, and 17,616 contigs were >1000 bp in length. 76% of the contigs were assembled within scaffolds with a mean length of 300 bp and 30,859 scaffolds were >1000 bp in length. The *A. mediterranea* Kmer 31 assembly yielded 675,534 contigs, with a mean length of 254 bp and N50 of 310 bp, and 16,755 contigs were >1000 bp in length. 82% of the contigs were assembled within scaffolds with a mean length of 301 bp and 26,884 scaffolds were >1000 bp in length.

### BLAST analysis of the assembled transcriptomes of *P. miniata*, *O. victoriae*, and *A. mediterranea*

To enable identification of transcripts encoding SALMFamide precursors in *P. miniata*, *O. victoriae*, and *A. mediterranea*, the contig and scaffold datasets generated from assembly of Illumina HiSeq sequence data obtained for these species were set up for BLAST (Basic Local Alignment Search Tool) analysis using SequenceServer^4^. BLAST searches were performed using the protein sequences of known SALMFamide precursors from *S. purpuratus*, *A. japonicus*, and *P. miniata* as queries (16). In addition, the sequence data were analysed by BLAST using short SALMFamide-type neuropeptide sequences as queries, with the *E* value set to 1000.

### Cloning and sequencing of SALMFamide precursor cDNAs from *O. victoriae* and *A. mediterranea*

Total RNA that had been generated for Illumina HiSeq sequencing (see above) was also used for cDNA synthesis (Quantitect Reverse Transcription Kit, QIAGEN, Manchester, UK). Full-length cDNAs of SALMFamide precursors, including 5′ and 3′ untranslated regions (UTR), were amplified through PCR (Phusion High-Fidelity PCR Master Mix, NEB, Hitchin, Hertfordshire, UK) using the oligos: 5′-GTGACATTACTACTCCTGAT-3′/5′-CAACAAGACAGACTAATGAC-3′(*O. victoriae* L-type SALMFamide precursor), 5′-GAAGTGGTTGCTAATACC-3′/5′-ACTTTAGTCCTTCCGTAC-3′(*O. victoriae* F-type SALMFamide precursor), and 5′-ATACAACGGGATAGAGAG-3′/5′-ACACTCGGAACTTGTCTA-3′(*A. mediterranea* SALMFamide precursor), designed using Primer3 software^5^. The PCR products were gel-extracted and purified (QIAquick Gel Extraction Kit, QIAGEN, Manchester, UK) before being blunt-end cloned into a pBluescript SKII (+) vector (Agilent Technologies, Stockport, Cheshire, UK) cut with the *Eco*RV-HF restriction endonuclease (NEB, Hitchin, Hertfordshire, UK). The clones were then sequenced (Eurofins Genomics GmbH, Ebersberg, Germany) from the T7 and T3 sequencing primer sites.

### Identification of SALMFamide precursors in other echinoderm species

Our efforts to determine the sequences of SALMFamide precursors in this study or in previous studies (16) have targeted a single species for each of the extant echinoderm classes: *S. purpuratus* (Echinoidea), *A. japonicus* (Holothuroidea), *P. miniata* (Asteroidea), *O. victoriae* (Ophiuroidea), and *A. mediterranea* (Crinoidea). To assess the generality of our findings, ideally transcriptome and/or genome sequence data from multiple species for each echinoderm class would be analysed. As a step toward this level of analysis, here we have analysed genome or transcriptome sequence data from at least one additional species from each of the five echinoderm classes.

To search for SALMFamide precursors in a second echinoid species, genome sequence data from the sea urchin *Lytechinus variegatus* was analysed using a BLAST facility^6^ made publicly available by Andy Cameron and colleagues at CalTech (USA). To search for SALMFamide precursors in species belonging to the four other echinoderm classes, transcriptome sequence data that have recently been obtained for an investigation of echinoderm phylogenetic relationships was analysed (21). The data analysed included transcriptome sequences from a holothurian species (*Leptosynapta tenuis*), an asteroid species (*Luidia senegalensis*), a crinoid species (*Aporometra wilsoni*), and 52 ophiuroid species (21).

## Results

### Determination of the sequences of transcripts encoding SALMFamide precursors in the starfish *P. miniata*

Previously, analysis of *P. miniata* genome sequence data enabled identification of two genes encoding SALMFamide-type neuropeptides in this species. Firstly, a gene encoding seven L-type SALMFamides (L-type gene) and secondly a gene encoding eight F-type SALMFamides and one L-type SALMFamide (F-type gene) (16). However, the predicted gene products reported by Elphick et al. (16) have yet to be confirmed by transcript sequencing. Therefore, here we analysed *P. miniata* transcriptome sequence data to identify SALMFamide precursor transcripts.

**Figure 1:**
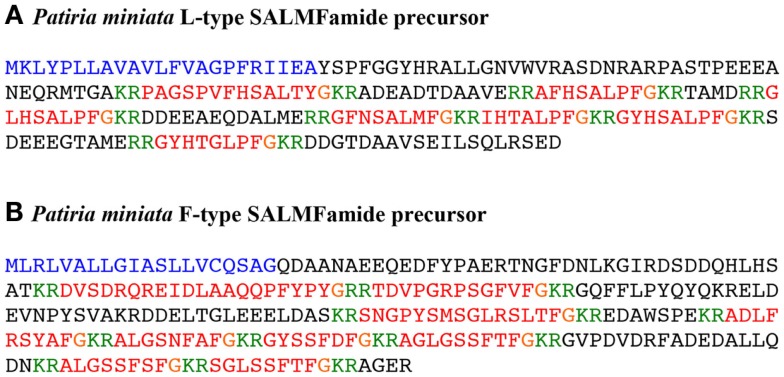
***Patiria miniata* SALMFamide precursors**. **(A)** L-type SALMFamide precursor. **(B)** F-type SALMFamide precursor. The predicted signal peptide of the precursor proteins is shown in blue and the putative SALMFamide neuropeptides are shown in red, with C-terminal glycine residues that are potential substrates for amidation shown in orange. Putative cleavage sites are shown in green.

BLAST analysis of *P. miniata* transcriptome data using the predicted 174-residue *P. miniata* L-type SALMFamide precursor sequence as a query identified a 1657 bp transcript (contig 378809) encoding a 212-residue protein (Figure 1A; Figure S1 in Supplementary Material). Residues 63–212 of this protein were found to be identical to residues 25–174 of the predicted 174-residue protein. However, the N-terminal region of the 212-residue protein (residues 1–62) did not share sequence identity with the N-terminal region of the predicted 174-residue protein (residues 1–24). Thus, the predicted 174-residue sequence of the L-type SALMFamide precursor in *P. miniata*, which was based on analysis of genomic sequence data, may be partially incorrect. Furthermore, BLAST analysis of *P. miniata* genome sequence data revealed that residues 1–62 of the 212-residue protein are encoded by an exon located on scaffold JH775329.1 (34230 bp), whereas residues 63–212 are encoded by an exon located on scaffold JH770521.1 (or 1914; 55,595 bp). Thus, the presence of the two exons on different genomic scaffolds provided an explanation for why the first exon was not identified when analysing genomic sequence data. The putative exon encoding residues 1–24 of the predicted 174-residue L-type SALMFamide precursor was identified on the same scaffold (JH770521.1) as the exon encoding the C-terminal region of the protein and was a plausible candidate exon because it encodes a polypeptide that has the expected properties for the N-terminal signal peptide region of a neuropeptide precursor. Transcriptome sequencing indicates that this prediction may have been wrong. However, there remains the possibility that this gene is subject to alternative splicing and transcripts encoding the predicted 174-residue L-type SALMFamide precursor occur naturally but are less abundant than transcripts encoding the 212-residue protein.

BLAST analysis using the predicted 258-residue *P. miniata* F-type SALMFamide precursor sequence as a query identified a 2041 base transcript (contig 387722) encoding a 258-residue protein that was identical to the query sequence (Figure 1B; Figure S2 in Supplementary Material). Thus, in this case analysis of transcriptome sequence data has provided confirmation of a sequence predicted from analysis of genome sequence data.

### Comparison of the structure of SALMFamide precursor genes in *S. purpuratus* and *P. miniata*

**Figure 2:**
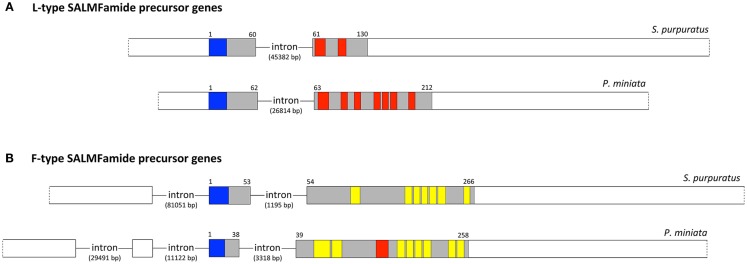
**Comparison of the structure of SALMFamide genes in the sea urchin *S. purpuratus* and the starfish *P. miniata***. **(A)**. Diagrammatic representation of the structure of the L-type SALMFamide precursor gene in *S. purpuratus* and *P. miniata*. **(B)**. Diagrammatic representation of the structure of the F-type SALMFamide precursor gene in *S. purpuratus* and *P. miniata*. Exons are drawn to scale as rectangles and introns are shown as lines but not to scale (intron lengths are shown in parentheses). Non-coding exons or regions of exons are shown in white; note that the 5′ and 3′ of the genes are shown as a dashed line because it is possible that additional non-coding bases are transcribed *in vivo* but are not present in transcripts that have been sequenced. The protein-coding exons or regions of exons are shaded (gray) or colored, with the N-terminal signal peptide shown in blue and the predicted neuropeptide products of the precursors shown in red (L-type SALMFamides) or yellow (F-type SALMFamides). The numbers above the protein-coding exons are amino-acid residue positions in the protein products of the genes. A feature that appears to distinguish L-type and F-type SALMFamide genes is the structure of the first protein-coding exons. Thus, in the two L-type SALMFamide genes the first protein-coding exon also encodes a 5′ non-coding region, whilst in the two F-type SALMFamide genes the first protein-coding exon does not have a 5′ non-coding region. These differences in gene structure provide important evidence that the L-type SALMFamide precursor genes in *S. purpuratus* and *P. miniata* are orthologs and the F-type SALMFamide precursor genes in *S. purpuratus* and *P. miniata* are orthologs.

Identification of transcripts encoding SALMFamide precursors in the starfish *P. miniata* (see above) has enabled analysis of the structure of the genes that encode these proteins and comparison with the structure of genes encoding SALMFamide precursors in the sea urchin *S. purpuratus*. Figure 2A shows a diagrammatic representation of the structure of genes encoding L-type SALMFamide precursors in *S. purpuratus* and *P. miniata* and Figure 2B shows a diagrammatic representation of the structure of genes encoding F-type SALMFamide precursors in *S. purpuratus* and *P. miniata*.

The L-type SALMFamide precursors in the two species have a similar gene structure comprising two exons. The first exon encodes a 5′ non-coding region and the N-terminal region of the precursor proteins, including the signal peptide. The second exon encodes the C-terminal region of the precursor proteins (including the L-type SALMFamides) and a long 3′ non-coding region.

The F-type SALMFamide precursors in the two species also have a similar gene structure but, importantly, the gene structure is distinctly different to the structure of the L-type SALMFamide genes. Thus, the F-type SALMFamide genes have one (*S. purpuratus*) or two (*P. miniata*) 5′ non-coding exons followed by an exon that encodes the N-terminal region of the precursor protein, with the first codon of the exon encoding the starter methionine. This contrasts with L-type SALMFamide precursor genes (see above), where the exon encoding the N-terminal region of the precursor also encodes a 5′ non-coding region. In common with L-type SALMFamide precursor genes, the C-terminal region of the F-type SALMFamide precursor proteins (which includes multiple copies of SALMFamide peptides) is encoded by a large exon with a long 3′ non-coding region.

Thus, L-type SALMFamide precursor and F-type SALMFamide precursor genes are similar in that the N-terminal and C-terminal regions of the proteins are encoded by two exons. However, the presence (L-type) or absence (F-type) of a 5′ non-coding region in the first protein-encoding exons is a distinguishing feature. This provides evidence that the L-type SALMFamide precursors in *S. purpuratus* and *P. miniata* are orthologous and F-type SALMFamide precursors in *S. purpuratus* and *P. miniata* are orthologous, which is an important finding because evidence for orthology based solely on comparison of protein sequences is not very strong, particularly for L-type SALMFamide precursors. Thus, whilst L-type SALMFamide precursors are characterized by the presence of peptides with a C-terminal SxLxLamide (L-type) motif, the number of copies of these peptides is very different in *P. miniata* (seven) and *S. purpuratus* (two). Indeed the presence of seven SALMFamides in the *P. miniata* L-type SALMFamide precursor makes this protein, at least superficially, more similar to F-type SALMFamide precursors in *P. miniata* and *S. purpuratus*, which contain nine and seven SALMFamides, respectively. In conclusion, therefore, the evidence of orthology provided by similarities/differences in gene structure provides an important basis for classifying SALMFamide genes in echinoderms as either L-type SALMFamide precursor genes or F-type SALMFamide precursor genes.

### Identification of an L-type SALMFamide precursor in the ophiuroid *O. victoriae*

**Figure 3:**
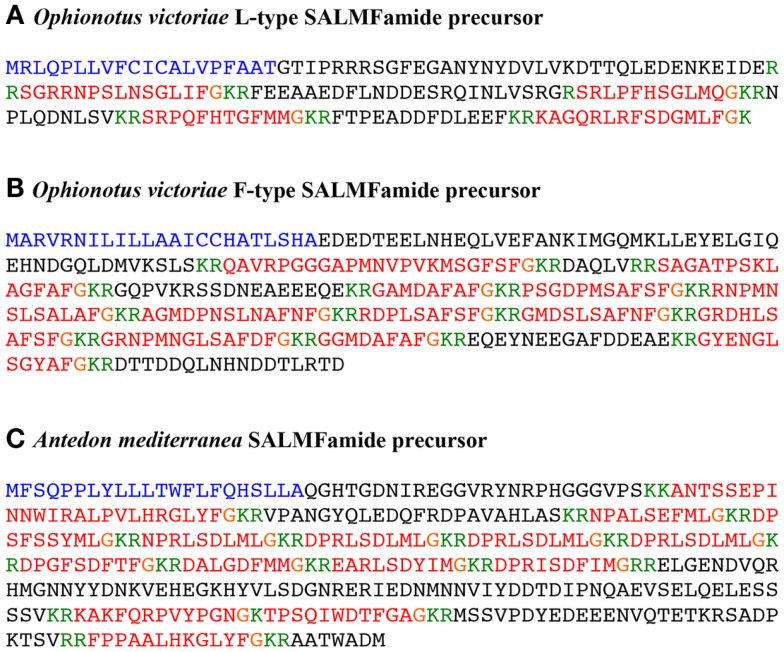
***Ophionotus victoriae* and *Antedon mediterranea* SALMFamide precursors**. **(A)** *O. victoriae* L-type SALMFamide precursor. **(B)** *O. victoriae* F-type SALMFamide precursor. **(C)** *A. mediterranea* SALMFamide precursor. The predicted signal peptide of the precursor proteins is shown in blue, putative neuropeptides derived from the precursors are shown in red with C-terminal glycine residues that likely substrates for amidation shown in orange. Putative cleavage sites are shown in green.

BLAST analysis of *O. victoriae* transcriptome sequence data revealed a 2422 bp contig (2059646) encoding a 169-residue protein comprising a predicted 20-residue N-terminal signal peptide and four putative SALMFamide neuropeptides bounded by monobasic or dibasic cleavage sites. A cDNA encoding this protein was cloned and sequenced, which confirmed the coding sequence predicted from assembled RNAseq data (Figure 3A; Figure S3 in Supplementary Material; GenBank accession number: KM979353). Analysis of the sequences of the four putative SALMFamide neuropeptides derived from this precursor revealed that the first (i.e., N-terminally located) is a dodecapeptide with a canonical L-type SALMFamide motif (SxLxFamide) and the fourth (i.e., C-terminally located) is a putative 14-residue neuropeptide with an L-type-like SALMFamide motif (SxMxFamide). The intervening second putative neuropeptide is an 11-residue peptide with the C-terminal pentapeptide sequence SGLMQamide, which is L-type-like based on the presence of the leucine residue but is otherwise very unusual in having a C-terminal glutamine residue. The third neuropeptide is also a putative 11-residue peptide but with the C-terminal pentapeptide sequence TGFMMamide, which is F-type-like based on the presence of the phenylalanine residue but it has C-terminal methionine residue. Thus, this is predominantly a precursor of L-type or L-type-like SALMFamide precursor, homologous with L-type SALMFamide precursors that have been identified in other echinoderms. However, it is atypical in containing an F-type-like SALMFamide with a C-terminal methionine.

### Identification of an F-type SALMFamide precursor in the ophiuroid *O. victoriae*

BLAST analysis of *O. victoriae* transcriptome sequence data revealed a 2343 bp scaffold (64,804) encoding a partial protein sequence comprising a predicted 23-residue N-terminal signal peptide and 11 putative SALMFamide neuropeptides bounded by dibasic cleavage sites. Because the protein-coding region of scaffold 64,804 was interrupted by a segment of unknown nucleotides it was necessary to clone and sequence a cDNA encoding this protein to determine its complete sequence. This revealed that it is a 310-residue protein comprising 12 putative SALMFamide neuropeptides, 11 of them F-type SALMFamides, and 1 of them an L-type SALMFamide (Figure 3B; Figure S4 in Supplementary Material; GenBank accession number: KM979352). Thus, this precursor protein is similar to the F-type SALMFamide precursor in the starfish *P. miniata*, which comprises eight F-type or F-type-like SALMFamides and one L-type SALMFamide [Figure 1; (16)].

### Identification of a SALMFamide precursor in the crinoid *A. mediterranea*

BLAST analysis of *A. mediterranea* transcriptome sequence data identified a putative 1683 bp transcript encoding a SALMFamide precursor protein, which was assembled manually from three overlapping contigs (1781194, 446850, and 1694464) and then confirmed by cDNA cloning and sequencing (Figure 3C; Figure S5 in Supplementary Material; GenBank accession number: KM979351). The SALMFamide precursor is a 370-residue protein comprising a predicted 22-residue N-terminal signal peptide and 12 putative SALMFamide-type neuropeptides bounded by dibasic cleavage sites. Two of the putative peptides have a C-terminal LxFamide motif (i.e., L-type SALMFamide) and one of the putative peptides has a C-terminal FxFamide motif (i.e., F-type). However, the other nine putative peptides have a variety of C-terminal motifs that include FxLamide, FxMamide, YxLamide, YxMamide, and LxLamide. The motif F/YxL/Mamide has previously been referred to as “F-type-like” and the motif LxLamide has previously been referred to as “L-type-like” (16). However, as discussed below, discovery of the *A. mediterranea* SALMFamide precursor may require reclassification of SALMFamide neuropeptides in echinoderms.

### Identification of SALMFamide precursors in other echinoderm species

Our findings from analysis of transcriptome or genome sequence data from other echinoderm species were consistent with those reported previously (16) or above, as described below and as illustrated in Figures S6–S10 in Supplementary Material.

An L-type SALMFamide precursor and an F-type SALMFamide precursor were identified in the sea urchin *Lytechinus variegatus* (class Echinoidea; Figure S6 in Supplementary Material), and as in *S. purpuratus*, the *L. variegatus* L-type SALMFamide precursor comprised two L-type SALMFamides (Figure S6A in Supplementary Material) and the *L. variegatus* F-type SALMFamide precursor comprised seven F-type SALMFamides (Figure S6B in Supplementary Material).

Interestingly, in the sea cucumber *Leptosynapta tenuis* (class Holothuroidea) two L-type SALMFamide precursors and one F-type SALMFamide precursor were identified (Figure S7 in Supplementary Material). Comparison of the sequences of the two L-type SALMFamide precursors in *L. tenuis* revealed high levels of sequence similarity (Figure S7C in Supplementary Material), indicating that these have arisen by gene duplication in this species or in a lineage that includes this species and other closely related holothurian species. As in the *A. japonicus* L-type SALMFamide precursor, both of the *L. tenuis* L-type SALMFamide precursors comprised three L-type or L-type-like SALMFamide neuropeptides (Figure S7A,B in Supplementary Material). The general characteristics of the F-type SALMFamide precursor in *L. tenuis* were similar to the F-type SALMFamide precursor in *A. japonicus* in being largely comprised of F-type SALMFamides. However, the overall level of sequence identity was quite low and in the positions occupied by two L-type SALMFamides in *A. japonicus* there are F-type SALMFamides in *L. tenuis*. Analysis of a wider range of species will be required to determine which of these represents the ancestral/derived condition.

In the starfish *Luidia senegalensis* (class Asteroidea), an L-type SALMFamide precursor and an F-type SALMFamide precursor were identified (Figure S8 in Supplementary Material). The *L. senegalensis* L-type SALMFamide precursor comprises seven L-type SALMFamides, which share high levels of sequence similarity with the seven corresponding L-type SALMFamides in the *P. miniata* L-type SALMFamide precursor (Figure S8A in Supplementary Material). The *L. senegalensis* F-type SALMFamide precursor comprises eight SALMFamides, six of which are F-type or F-type-like, and two of which are L-type. By way of comparison, the *P. miniata* F-type SALMFamide precursor comprises nine SALMFamides, eight F-type, and one L-type (Figure S8B in Supplementary Material). Thus, whilst the overall organization of the F-type SALMFamide precursors in the two species is similar and clearly indicative of orthology, lineage specific gain/loss of peptides has occurred as well as conversion of peptides from F-type to L-type or vice versa. Analysis of a wider range of species will therefore be necessary to identify the ancestral characteristics of F-type SALMFamide precursors in asteroids.

Recent molecular phylogenetic analysis of the class Ophiuroidea has identified three clades: clade A, clade B, and clade C (21). Here, an L-type SALMFamide precursor and an F-type SALMFamide precursor were identified in species belonging to each of these clades. In clade A, which includes *O. victoriae*, these were *Ophiomusium lymani* (Ophiolepididae), *Asteronyx loveni* (Asteronychidae), and *Asteroschema bidwillae* (Asterochematidae). In clade B *Clarkoma canaliculata* (Ophiocomidae) and in clade C *Ophiactis abyssicola* (Ophiactidae) and *Ophiothrix angulata* (Ophiotrichidae). The characteristics of the L-type SALMFamide and F-type SALMFamide precursor in these species were similar to those in *O. victoriae*, and this is illustrated using sequence data from *Ophiothrix angulata* as an example in Figure S9 in Supplementary Material. Thus, the L-type SALMFamide precursors in *O. victoriae* and *O. angulata* comprise four homologous peptides. However, as reported above for other classes, there is variable conservation of the sequences of peptides that occupy equivalent positions in the precursor proteins. For example, the C-terminal region of the first SALMFamide in the precursor is very similar in both species (LNSGLxFamide), whereas the third SALMFamide exhibits sequence divergence – TGFMMamide in *O. victoriae* and SAMLLamide *O. angulata* (Figure S9A in Supplementary Material). The F-type SALMFamide precursor in *O. angulata* comprises 10 F-type SALMFamides and 1 L-type SALMFamide. This is similar to the F-type precursor in *O. victoriae*, which comprises 11 F-type SALMFamides and 1 L-type SALMFamide, with the L-type SALMFamide occupying the same position in both species (Figure S9B in Supplementary Material).

In the feather star *Apotometra wilsoni* (class Crinoidea), only a single SALMFamide precursor was identified, consistent with our findings from *A. mediterranea* (Figure S10 in Supplementary Material). Comparison of the sequences of the SALMFamide precursor in *A. mediterranea* and *A. wilsoni* revealed high levels of similarity, with both precursors comprising 14 putative SALMFamide neuropeptides.

An example where there is sequence divergence is the seventh peptide, which has the C-terminal sequence LMLamide in *A. mediterranea* and FMLamide in *A. wilsoni*. However, peptides occupying equivalent positions in the two precursors are largely similar in their sequence characteristics.

## Discussion

Previously, analysis of genome/transcriptome sequence data has enabled identification of genes/transcripts encoding SALMFamide neuropeptide precursors in species from three of the five extant echinoderm classes: the sea urchin *S. purpuratus* (Echinoidea), the sea cucumber *A. japonicus* (Holothuroidea), and the starfish *P. miniata* (Asteroidea) (16). This has revealed that in each species there are two SALMFamide precursor genes: firstly, a gene that encodes peptides with a C-terminal LxFamide-type motif (L-type) and secondly, a gene that either only encodes peptides with a C-terminal FxFamide-type motif (F-type) (*S. purpuratus*) or encodes F-type SALMFamides plus one or more L-type SALMFamides (*A. japonicus*, *P. miniata*) (16). However, the *P. miniata* sequences were based on predictions made from genome sequence data, without supporting evidence from transcript sequences. Furthermore, a deeper understanding of SALMFamide precursor evolution in the phylum Echinodermata will require analysis of sequence data from at least one species from the two other extant echinoderm classes – the Ophiuroidea and Crinoidea.

Here, we have determined the sequences of transcripts encoding the L-type SALMFamide precursor and F-type SALMFamide precursor in the starfish *P. miniata*, which has enabled comparison of the structure of genes encoding these precursors with SALMFamide precursor genes in the sea urchin *S. purpuratus*. This has revealed that F-type SALMFamide precursor genes have one or two introns in the 5′ non-coding region of the genes, whereas L-type SALMFamide genes do not have this feature. The overall level of sequence identity when comparing L-type SALMFamide precursors or F-type SALMFamide precursors protein sequences from different echinoderm classes is low [see Figures 5 and 6 in Ref. (16)]. Consequently, the categorization of SALMFamide precursors into two types (L-type and F-type) has hitherto been based upon the relative abundance of constituent peptides that have either an LxFamide motif or FxFamide motif. Obtaining evidence of orthology based on gene structure now provides an important additional criterion for classification of SALMFamide precursors as either L-type or F-type.

The F-type SALMFamide precursors in *A. japonicus* (echinozoan clade) and *P. miniata* (asterozoan clade) are largely comprised of F-type SALMFamides but they also contain one or more L-type SALMFamides. This suggests that the occurrence of L-type SALMFamides in F-type precursors may be a characteristic of F-type precursors traceable to the common ancestor of the Asterozoa and Echinozoa, with the absence of L-type SALMFamides in the F-type precursors of sea urchins (Echinoidea, Echinozoa) presumably a derived feature. Here, we have obtained evidence in support of this hypothesis with the identification of a transcript encoding a F-type precursor in the brittle star *O. victoriae* (Ophiuroidea, Asterozoa). Thus, in *O. victoriae* there is an F-type precursor that largely comprises F-type SALMFamides (11) but which also contains an L-type SALMFamide. Similarly, the F-type precursor in the brittle star *O. angulata* comprises 10 F-type SALMFamides and 1 L-type SALMFamide. By way of comparison, the L-type SALMFamide precursors in *O. victoriae* and *O. angulata* comprise four putative peptides, including two L-type SALMFamides and an unusual peptide with a C-terminal LxQamide motif. This latter feature is atypical of L-type precursors that have been identified in other echinoderms and therefore it appears to be derived characteristic of ophiuroids.

Analysis of transcriptome sequence data from the crinoid *A. mediterranea* revealed only a single SALMFamide-type precursor. A homolog of this precursor was found in the feather star *A. wilsoni* and, as in *A. mediterranea*, this was the only SALMFamide precursor identified in this species. Analysis of the sequences of the putative neuropeptides derived from the *A. mediterranea* precursor reveals that it comprises five peptides with a FxLamide-type motif and four peptides with a LxLamide-type motif but only two peptides with a LxFamide-type motif and one peptide with a FxFamide-type motif. Based upon these characteristics, the *A. mediterranea* SALMFamide precursor does not appear to be an L-type-like precursor, which are largely comprised of peptides with a LxFamide motif, or an F-type-like precursor, which are largely comprised of peptides with a FxFamide motif. Thus, discovery of SALMFamide precursors in crinoid species has broadened our perspective on the structural characteristics of SALMFamides.

**Figure 4:**
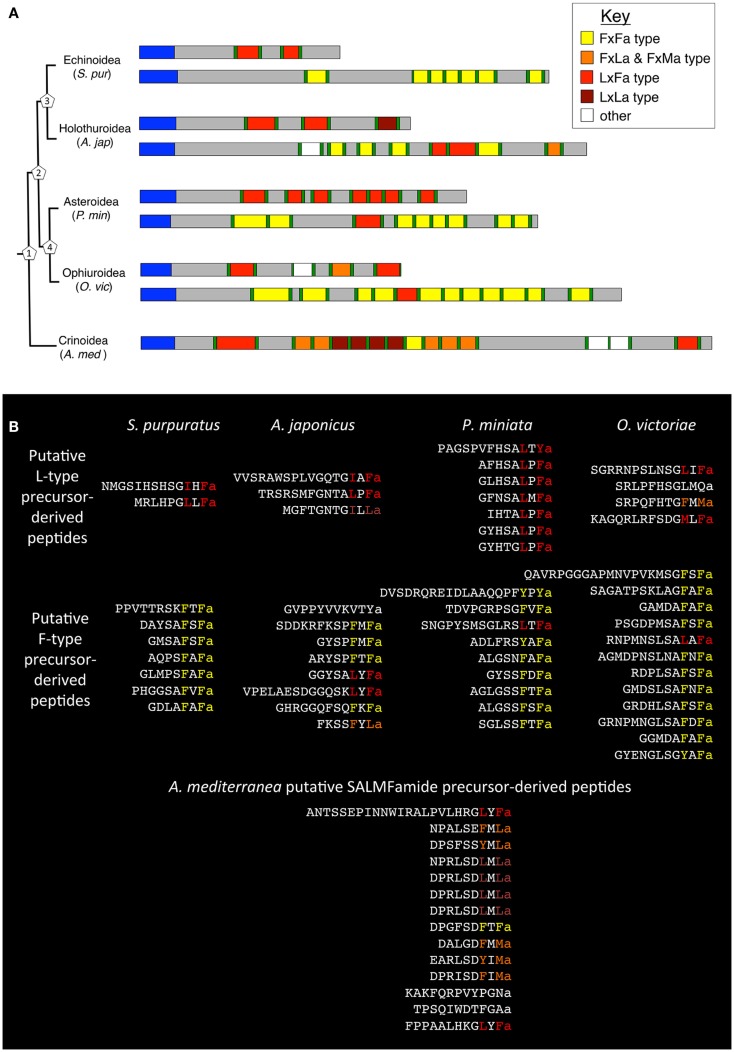
**The occurrence and properties of SALMFamide precursors (A) and putative SALMFamide peptides (B) in species representing each of the five extant echinoderm classes**. **(A)** SALMFamide precursors are shown in a phylogenetic diagram according to the phylogeny of Telford et al. (18) and O’Hara et al. (21), with crinoids basal to the Echinozoa (Holothuroidea and Echinoidea) and the Asterozoa (Asteriodea + Ophiuroidea). The estimated divergence times for the nodes (labeled with numbers in pentagons) according to O’Hara et al. (21) are: 1. 501-542 Ma, 2. 482-421 Ma, 3. 464-485 Ma, and 4. at least 479 Ma. *S. pur* is the sea urchin *Strongylocentrotus purpuratus* (Echinoidea), *A. jap* is the sea cucumber *Apostichopus japonicus* (Holothuroidea), *P. min* is the starfish *Patiria miniata* (Asteroidea), *O. vic* is the brittle star *Ophionotus victoriae* (Ophiuroidea), and *A. med* is the feather star *Antedon mediterranea* (Crinoidea). Signal peptides are shown in blue and dibasic or monobasic cleavage sites are shown in green. L-type SALMFamides with a C-terminal LxFamide motif or with an L-type-like motif (e.g., IxFamide) are shown in red. F-type SALMFamides with a FxFamide motif or with an F-type-like motif (e.g., YxFamide) are shown in yellow. SALMFamides with a FxLamide-type motif are shown in orange and SALMFamides with a LxLamide motif are shown in dark red. Peptides that do not conform with any of the four color-coded categories are shown in white (e.g., GVPPYVVKVTYamide in *A. japonicus* and SRLPFHSGLMQamide in *O. victoriae*). The diagram shows how in a presumed ancestral-type precursor in crinoids the majority of the putative peptides have a FxLamide-type motif or a LxLamide-type motif and there is only one L-type SALMFamide and one F-type SALMFamide. However, as a consequence of specialization following a presumed duplication of the ancestral-type gene in a common ancestor of the Echinozoa and Asterozoa, two types of SALMFamide precursor have evolved: one that is predominantly comprised of L-type SALMFamides (red) and another that is exclusively or predominantly comprised of F-type SALMFamides (yellow). **(B)** C-terminal alignments of SALMFamide neuropeptides derived from the precursor proteins shown in **(A)**. The C-terminal regions of each peptide are color-coded according to the key shown in **(A)**.

Hitherto SALMFamides have been characterized as either L-type (LxFamide) or F-type (FxFamide). Peptides that deviate from these motifs have been identified in echinozoans and asterozoans; for example, one of the peptides in the *A. japonicus* L-type SALMFamide precursor has the C-terminal motif IxLamide, which previously was categorized as L-type-like. Similarly, one of the peptides in the *A. japonicus* F-type SALMFamide precursor has the C-terminal motif FxLamide, which was categorized as F-type-like. Our discovery that the crinoid SALMFamide precursor largely comprises peptides with a LxLamide motif or LxLamide-like motif (e.g., LxMamide) and peptides with a FxLamide motif has provided a basis for categorization of SALMFamides into four types: 1. FxFamide, 2. FxLamide or FxMamide, 3. LxFamide, and 4. LxLamide (Figure 4). However, we retain the use of the terms L-type (LxFamide) and F-type (FxFamide) because these are useful for categorization of SALMFamide peptides and SALMFamide precursors in echinozoans and asterozoans. Furthermore, identification of transcripts encoding SALMFamide precursors in species representing all five extant echinoderm classes, the feather stars *A. mediterranea* and *A. wilsoni* (Crinoidea), the brittle stars *O. victoriae* and *O. angulata* (Ophiuroidea), the starfishes *P. miniata* and *L. senegalensis* (Asteroidea), the sea cucumbers *A. japonicus* and *L. tenuis* (Holothuroidea), and the sea urchins *S. purpuratus* and *L. variegatus* (Echinoidea), provides a basis for formulation of hypotheses on the evolution of these proteins.

Because crinoids are basal to the Echinozoa (Echinoidea and Holothuroidea) and the Asterozoa (Asteroidea and Ophiuroidea) in echinoderm phylogeny, one model of SALMFamide precursor evolution in the phylum Echinodermata is that ancestrally there was a single SALMFamide gene encoding a variety of SALMFamides (as in *A. mediterranea* and *A. wilsoni*), which duplicated in a common ancestor of the Echinozoa and Asterozoa. Then one of the duplicated genes specialized to encode L-type SALMFamides and the other specialized to exclusively or predominantly encode F-type SALMFamides (as in Echinozoa and Asterozoa). In the context of this evolutionary scenario, it is of interest to compare the structural features of SALMFamide precursors in the five echinoderm classes, using the precursors identified in *S. purpuratus*, *A. japonicus*, *P. miniata*, *O. victoriae*, and *A. mediterranea* as examples (see Figure 4A). Starting with the single SALMFamide precursor identified in the crinoid *A. mediterranea* and with reference to the key, which shows the color coding for the different types of SALMFamides, the predominance of peptides with a FxLamide-type or FxMamide-type motif (orange) or a LxLamide-type motif (dark red) is apparent, whilst the prototypical L-type SALMFamides (LxFamide; red) and F-type SALMFamides (FxFamide; yellow) are minor components. What cannot be deduced from this single precursor in an extant crinoid is whether or not this precursor structure is representative of an ancestral-type SALMFamide precursor that would have existed in extinct crinoid species ~550 million years ago, before the emergence of echinozoan and asterozoan echinoderms (18, 21, 22). In the lineage leading to extant feather stars (order Comatulidina; infraorder Comatulidia) such as *A. mediterranea*, intragenic duplication events may have resulted in expansion of segments of DNA encoding some SALMFamide neuropeptide types, whilst mutational changes or deletion events may have resulted in loss of other SALMFamide neuropeptide types. Further insight on this issue may be obtained if SALMFamide precursor transcript/gene sequences are obtained in the future with broader taxonomic sampling of extant crinoids. Nevertheless, we speculate that the occurrence of multiple types of SALMFamides (FxFamide-type, FxLamide-type or FxMamide-type, LxFamide-type, and LxLamide-type) in the *A. mediterranea* SALMFamide precursor is reflective of the ancestral condition in extinct crinoids that predated the emergence of asterozoans and echinozoans, although of course the precise number of copies of each type may be variable between extant crinoid species.

If the *A. mediterranea* precursor is representative of an ancestral-type SALMFamide precursor in being comprised of a variety of SALMFamides that have C-terminal FxFamide-type, FxLamide-type or FxMamide-type, LxFamide-type, and LxLamide-type motifs, then the occurrence of SALMFamide precursors in asterozoans and echinozoans that are exclusively or predominantly comprised of peptides with a LxFamide-type motif (L-type precursor) or FxFamide-type motif (F-type precursor) is interesting from both an evolutionary and functional perspective. It suggests that following the putative duplication of a gene encoding an ancestral-type SALMFamide precursor in a common ancestor of echinozoans and asterozoans, there was specialization toward on the one hand a precursor of L-type peptides (LxFamide) and on the other hand a precursor of F-type peptides (FxFamide). Conversely, peptides with a LxLamide-type motif, FxLamide-type motif, or FxMamide-type motif that predominate in the *A. mediterranea* precursor are either absent or are minor components of SALMFamide precursors in echinozoans and asterozoans (see Figure 4B). This specialization of SALMFamide precursors as sources of either L-type or F-type SALMFamide neuropeptides would presumably reflect acquisition of distinct physiological roles. Therefore, it will be of interest to compare the patterns of expression of L-type precursors and F-type precursors and the pharmacological actions of their constituent neuropeptides in echinozoan and asterozoan species.

An alternative hypothesis to that outlined above is that a second SALMFamide-type precursor remains to be identified in crinoid species and that it remains undiscovered because of sequence divergence or incomplete transcriptome data. Another scenario is that two SALMFamide-type precursors existed ancestrally in crinoids but there has been loss of one precursor in a lineage that gave rise to extant feather star species such as *A. mediterranea* and *A. wilsoni*. If either of these scenarios is correct, then the question arises as to the relationship of the crinoid SALMFamide-type precursor identified here with the L-type and F-type SALMFamide precursors in asterozoa and echinozoa. Based on the number of constituent peptides, the crinoid SALMFamide-type precursor identified here is more similar to F-type SALMFamide precursors than L-type SALMFamide precursors. Further insights on the evolutionary relationships of SALMFamide neuropeptides and their precursors will be gained if the receptors that mediate their effects are identified.

## Author Contributions

This study was conceived and co-ordinated by Maurice R. Elphick. *P. miniata* transcriptome sequence data were obtained by Judith Levine and Christopher J. Lowe. *O. victoriae* and *A. mediterranea* transcriptome sequence data were obtained by Melody S. Clark, Maria Ina. Arnone, Dean C. Semmens, Liisa M. Blowes, and Maurice R. Elphick. Analysis of *P. miniata, O. victoriae*, and *A. mediterranea* transcriptome sequence data was performed by Maurice R. Elphick, Dean C. Semmens, and Liisa M. Blowes. Cloning and sequencing of the *O. victoriae* and *A. mediterranea* SALMFamide precursor cDNAs was performed by Dean C. Semmens. The manuscript was written by Maurice R. Elphick and Dean C. Semmens, with all other authors editing or commenting on the final draft of the manuscript.

## Conflict of Interest Statement

The authors declare that the research was conducted in the absence of any commercial or financial relationships that could be construed as a potential conflict of interest.

## Supplementary Material

The Supplementary Material for this article can be found online at http://www.frontiersin.org/Journal/10.3389/fendo.2015.00002/abstract

## Footnotes

^1^http://www.bioinformatics.babraham.ac.uk/projects/fastqc/

^2^http://soap.genomics.org.cn/SOAPdenovo-Trans.html

^3^www.hpc.qmul.ac.uk

^4^http://www.sequenceserver.com

^5^http://bioinfo.ut.ee/primer3-0.4.0/

^6^http://www.echinobase.org/Echinobase/Blast/LvBlast/blast.php
