# Supplementary material for: Reconstructing SALMFamide Neuropeptide Precursor Evolution in the Phylum Echinodermata: Ophiuroid and Crinoid Sequence Data Provide New Insights
Source: Front Endocrinol (Lausanne). 2015 Feb 2;6:2. doi: 10.3389/fendo.2015.00002 (PMC4313774; doi:10.3389/fendo.2015.00002)
Supplement: Supplementary file 1 [file Presentation_1.ZIP › Figure S1.PDF]

```
1                                                              g
2    tagccacttgacacgccgttttgtcacttggtcccgctctatttctcgcagcggtacgag
62   ccagactgagcttactcagcattatcaccgggacaagagctgccgagtccacggggctga
122  cgacggggatttccctggttgggttgttggtttttttggttttgattcgcggtgaatggtc
182  cgagttatcccgatgaagttgtaccctcttctcgccgtagcggtgttgttcgtcgccggt
                 M  K  L  Y  P  L  L  A  V  A  V  L  F  V  A  G    16
242  ccgttcaggatcatcgaggcatactcgccctttggcggctaccaccgagcgctgctgggc
      P  F  R  I  I  E  A  Y  S  P  F  G  G  Y  H  R  A  L  L  G   36
302  aatgtgtgggttcgagcgtcggacaacagggcccgacctgcatcgacgccagaggaagaa
      N  V  W  V  R  A  S  D  N  R  A  R  P  A  S  T  P  E  E  E   56
362  gctaacgaacagagaatgacgggtgccaagcgacccgccggttcgcccgtgtttcactcg
      A  N  E  Q  R  M  T  G  A  K  R  P  A  G  S  P  V  F  H  S   76
422  gctctgacgtacggcaagcgagccgacgaagcagacactgatgccgcagtggagcgaaga
      A  L  T  Y  G  K  R  A  D  E  A  D  T  D  A  A  V  E  R  R   92
482  gctttccattccgccttacccttcggcaagagaacagccatggacagacggggactgcac
      A  F  H  S  A  L  P  F  G  K  R  T  A  M  D  R  R  G  L  H  116
542  agcgccctccccttcggtaaacgcgacgacgaagaggccgagcaggacgccttgatggag
      S  A  L  P  F  G  K  R  D  D  E  E  A  E  Q  D  A  L  M  E  136
602  aggaggggtttcaactcggccctgatgttcggcaaacggatacacaccgccttgccgttc
      R  R  G  F  N  S  A  L  M  F  G  K  R  I  H  T  A  L  P  F  156
662  ggcaagcggggttaccacagcgctttgccgttcgggaagcgttcggacgaggaggaaggc
      G  K  R  G  Y  H  S  A  L  P  F  G  K  R  S  D  E  E  E  G  176
722  actgcgatggagcggagaggctaccacaccggcctgccgttcggcaagcgagacgacggg
      T  A  M  E  R  R  G  Y  H  T  G  L  P  F  G  K  R  D  D  G  296
782  accgacgcagcggtcagtgagatattaagtcaactacggagtgaagattaaggggaaaa
      T  D  A  A  V  S  E  I  L  S  Q  L  R  S  E  D  *           212
842  actgacactttaaaaaaaggacgttagaattactagctacaatacacaaaatgatcaatg
902  tgcaatggtgactcttgctttgggttttacattcgtttcatctgcgaaaccgaacgcgtt
962  cgtgtgtctttcaaaccgagaaagatcattgtgacactaattatggacaaccaaatattt
1022 cctttttttctttgagtatacggtctcaatttgatgtaggtttagtttttgtttgggtgt
1082 tcataccatttcatgacttcgtgtttggtttaatgtctgggttctggcaattgaaaatgg
1142 gattaaattaatcaaggtaaaattaatgataatagtgcctgctaaaaacaaacgctgatt
1202 aatgtagttgtgaaatgacaaatattgatagcttttcgtgcaccgtctgatttttcgcac
1262 tgtgaacacactgaacaaatgtcagctctgcacctaatagatgatatcgaaagtaagaaa
1322 cagacaatatttagcttaattacatatttcttttgaattaacagtacactgccattaacc
1382 taaatttaatctattatgagtgatattacgttaccagattttttttcttttttacaagtt
1442 ttaacctgaaccaatcagtgtgaaacacagtagcacgtaaaaccacccgccccaacggct
1502 gttagatgcattcagtggcgtatatccgtgttggcgagtgggggggatgggggacaatggc
1562 ggatccagaacacacatttttttgggggggggggccaaaacaaaaaatttggtccccaaa
1622 aagtgggtccaaaaaataaaagtgtggggttcgggg
```

Figure S1. ***Patiria miniata*** **L-type SALMFamide precursor.** The sequence of a 1657 bp transcript (contig 378809) in *P. miniata* that encodes an L-type SALMFamide precursor protein (bold uppercase, 212 amino acid residues) is shown. The predicted signal peptide of the precursor protein is shown in blue and the seven putative SALMFamide neuropeptides are shown in red, with C-terminal glycine residues that are potential substrates for amidation shown in orange. Putative dibasic cleavage sites (KR or RR) are shown in green. The asterisk shows the position of the stop codon.
