# Supplementary material for: Reconstructing SALMFamide Neuropeptide Precursor Evolution in the Phylum Echinodermata: Ophiuroid and Crinoid Sequence Data Provide New Insights
Source: Front Endocrinol (Lausanne). 2015 Feb 2;6:2. doi: 10.3389/fendo.2015.00002 (PMC4313774; doi:10.3389/fendo.2015.00002)
Supplement: Supplementary file 1 [file Presentation_1.ZIP › Figure S10.PDF]

```
BLAST Query = A. mediterranea SALMFamide precursor (370 letters)
Database = Aporometra wilsoni transcriptome
Hit = comp29396_c0_seq1

A. med: 1     MFSQPPLYLLLTWFLFQHSLLAQGHTGDNIREGGVRYNRPHGGGVPSKKANTSSEPINNW  60
              MFSQ PL LLL+W + QHSLL  GH GD +R+G +RYNRP G  +PSKKAN  S PINNW
A. wil: 1     MFSQSPLLLLLSWIILQHSLLTLGHAGDKLRDGAMRYNRPIG--MPSKKANNDSAPINNW  58

A. med: 61    IRALPVLHRGLYFGKRVPANGY-QLEDQ--FRDPAVAHLASKRNPALSEFMLGKRDPSFS  117
              IRALP+LH+GL+FGKR+PANG+ Q E Q  FR+  + H ASKR+PA S+FMLGKRDP  S
A. wil: 59    IRALPLLHQGLFFGKRIPANGFIQDEGQKNFRNSLLGHWASKRDPAFSDFMLGKRDPRLS  118

A. med: 118   SYMLGKRNPRLSDLMLGKRDPRLSDLMLGKRDPRLSDLMLGKRDPRLSDLMLGKRDPGFS  177
                MLGKR+PRLSDLMLGKRDPR+SDLMLGKRDPRLSDLMLGKRDP  SD MLGKRDP FS
A. wil: 119   DLMLGKRDPRLSDLMLGKRDPRISDLMLGKRDPRLSDLMLGKRDPAFSDFMLGKRDPRFS  178

A. med: 178   DFTFGKRD-ALGDFMMGKREARLSDYIMGKRDPRISDFIMGRREL--GENDVQRHMGNNY  234
              +F  GKRD  L DFMMGKREARLSD+IMGKRDPR+S+F+MGRREL  GE++  R   NNY
A. wil: 179   EFMLGKRDPLLSDFMMGKREARLSDFIMGKRDPRLSEFMMGRRELGIGEHETGRQHANNY  238

A. med: 235   YDNKVEHEGKHYVLSDGNR-ERIEDNMNNVIYDDTDIPNQAEVSELQELESSSSVKRKAK  293
              YDN+VEH+    YV+SD +    R+E+NM N+IYDDTDIPN+AE   E+Q+LESSSS+KRK K
A. wil: 239   YDNEVEHDANRYVVSDQHLGRRLEENMGNIIYDDTDIPNEAEAPEMQDLESSSSLKRKVK  298

A. med: 294   FQRPVYPGNGKTPSQIWDTFGAGKRMSSVPDYEDEEEN--VQTETKRSAD--PKTSVRRF  349
                RP + G+GKTPS +W+ FGAGKRMSSV DYEDEEEN  VQT  KRSAD  PKTSVRRF
A. wil: 299   IHRPSFTGSGKTPSVLWNNFGAGKRMSSVSDYEDEEENVPVQTNIKRSADPAPKTSVRRF  358

A. med: 350   PPAALHKGLYFGKRAATWADM  370
              PPAALHKGLYFGKR A WADM
A. wil: 359   PPAALHKGLYFGKREAAWADM  379
```

Figure S10 BLAST analysis of transcriptome sequence data from the sea cucumber *Aporometra wilsoni* (O'Hara et al., 2014) identifies a homolog of the *A. mediterranea* SALMFamide precursor. The putative SALMFamide neuropeptides are shown in red, with C-terminal glycine residues that likely substrates for amidation shown in orange, and putative cleavage sites are shown in green. In *A. mediterranea* there are fourteen putative SALMFamides and homologs of all of these are also present in *A. wilsoni.*
