# Supplementary material for: Reconstructing SALMFamide Neuropeptide Precursor Evolution in the Phylum Echinodermata: Ophiuroid and Crinoid Sequence Data Provide New Insights
Source: Front Endocrinol (Lausanne). 2015 Feb 2;6:2. doi: 10.3389/fendo.2015.00002 (PMC4313774; doi:10.3389/fendo.2015.00002)
Supplement: Supplementary file 1 [file Presentation_1.ZIP › Figure S2.PDF]

```
1                                                              gc
3    ctcgccaaagccgaacgtctcataagggcctctgcgtgcattgctttagctcagactggg
63   aggcaaacctctttgcgcacggacgaactcattctccagtgttgtgccactttgaaaagc
123  agtcgagccgtaaatcggtgttcaccaactgccttcccagccaagtggaagaaatcgctg
183  gctttggaagtttcactgcgggtgcggttttgtgttattcttttgcaagcggaagacata
243  gtttttggctgtattacatacaagtacgtcactgaagattgtgacagtgaaggaaatcg
303  agtcaccgttgaaaaaaagatcagagacgtcacaggggaaattttcagttaaagatgttg
                                                           M  L    2
363  cgactcgtagcccttcttggaatagctagtctgctagtatgccagtcggccggtcaggat
      R  L  V  A  L  L  G  I  A  S  L  L  V  C  Q  S  A  G  Q  D   22
423  gctgcaaacgcggaggaacaagaagactttaccctgctgagaggacgaatggttttgac
      A  A  N  A  E  E  Q  E  D  F  Y  P  A  E  R  T  N  G  F  D   42
483  aatttaaaaggaatcagggacagtgatgaccagcatctacacagcgccaccaagcgagat
      N  L  K  G  I  R  D  S  D  D  Q  H  L  H  S  A  T  K  R  D   62
543  gttagcgacaggcaacgtgagattgatctggcagcgcagcagccattctacccgtacggc
      V  S  D  R  Q  R  E  I  D  L  A  A  Q  Q  P  F  Y  P  Y  G   82
603  aggagaacagacgtaccgggccggcccagcggcttcgtattcggcaagagagggcaattc
      R  R  T  D  V  P  G  R  P  S  G  F  V  F  G  K  R  G  Q  F  102
663  ttcctcccttaccagtatcaaaagcgggaactagatgaggtcaacccgtacagcgtagcc
      F  L  P  Y  Q  Y  Q  K  R  E  L  D  E  V  N  P  Y  S  V  A  122
723  aagcgagacgacgagttgaccggtctggaggaggagcttgacgccagcaaacggtccaat
      K  R  D  D  E  L  T  G  L  E  E  E  L  D  A  S  K  R  S  N  142
783  ggaccgtatagcatgagcgggctgcgctcgctcactttcggcaagcgtgaagacgcgtgg
      G  P  Y  S  M  S  G  L  R  S  L  T  F  G  K  R  E  D  A  W  162
843  agcccggaaaagagagccgatctgttccgctcctacgcattcggcaagcgggcccttggc
      S  P  E  K  R  A  D  L  F  R  S  Y  A  F  G  K  R  A  L  G  182
903  agcaactttgccttcggcaagcgtggctactcctccttcgacttcggcaaacgcgcggga
      S  N  F  A  F  G  K  R  G  Y  S  S  F  D  F  G  K  R  A  G  202
963  ctcggcagcagctttaccttcggcaagagaggggtcccggatgtggacagattcgccgat
      L  G  S  S  F  T  F  G  K  R  G  V  P  D  V  D  R  F  A  D  222
1023 gaggacgccctcctgcaggacaacaagcgcgccctcggcagcagcttctcgttcggcaag
      E  D  A  L  L  Q  D  N  K  R  A  L  G  S  S  F  S  F  G  K  242
1083 cggagcggtctgtccagcttcaccttcggcaagcgggcgggtgaacgatagaacactgag
      R  S  G  L  S  S  F  T  F  G  K  R  A  G  E  R  *           258
1143 ggcgtcctatcgcatacccaacacttgtaacagattcactattattcttattatttcaac
1203 tcacgtttgattgaccactggtttatgtttttacgaaaagaccagaatgctggtcattca
1263 gtttttagccttaaaatagtttagccctgtcaagggacatttaatacaggagagagaaaa
1323 aaaggagaaaactaaacagttgaccatggaatacgtttgtaaataaagataacagttaca
1383 atctatactagaaaatgacgaagatttcttgtaagaaaggtcattaaaatttatgaccaa
1443 aatatattttctttttttcctttttaaagttttcgccaaattttcagatcccttaaatagtg
1503 tttctactcgggagttatgttatagctatgtgatgataaagtacttctttatttgcgtac
1563 tttggataaactatgaagtgcaaattgcacatatttggcaaatcatccaattgtatttat
1623 ttacttcattaataatagttacagtagaaggtgttaaagcaaacaacagtaaaatcaaga
1683 cggatttaatgtatttactttaggcttgatgatttttattaaagtcggttacgagacatt
1743 aatcctattctagtcggactttattgtaattaccagaagaaaaaaacccaacctgttgtg
1803 tgaaatcaatgtgtcgaaagcaaatgttagtggacaacttaaaaacatgaaatgccttag
1863 aaaggatagattaataaagacaaaaaatgcattccatgctagcgttttaatcaagagatg
1923 gaagtaaattatttttttccttccagatccgatttgtacataaaaaaaatcacaaaatatc
1983 aactgctagactttgtgaaattctgcgaaataaaatgatttgacttctgaggaaaaaaa
```

Figure S2. ***Patiria miniata*** **F-type SALMFamide precursor.** The sequence of a 2041 bp transcript (contig 387722) in *P. miniata* that encodes an F-type SALMFamide precursor protein (bold uppercase, 258 amino acid residues) is shown. The predicted signal peptide of the precursor protein is shown in blue and the seven putative SALMFamide neuropeptides are shown in red, with C-terminal glycine residues that are potential substrates for amidation shown in orange. Putative dibasic cleavage sites (KR or RR) are shown in green. The asterisk shows the position of the stop codon.
