# Supplementary material for: Reconstructing SALMFamide Neuropeptide Precursor Evolution in the Phylum Echinodermata: Ophiuroid and Crinoid Sequence Data Provide New Insights
Source: Front Endocrinol (Lausanne). 2015 Feb 2;6:2. doi: 10.3389/fendo.2015.00002 (PMC4313774; doi:10.3389/fendo.2015.00002)
Supplement: Supplementary file 1 [file Presentation_1.ZIP › Figure S3.PDF]

1 cc
3 gtcagaggttggagtctcatctgcaaacaacaggtgccgtgaagcagacgacacaacgag
63 agctttcaatcg**gtgacattactactcctgat**tttggctccaattttctgttattttgt
123 ttccagatgagactccaacctctgcttgtgttttgtatatgtgcgctagtaccatttgct
M R L Q P L L V F C I C A L V P F A 18
183 gctacagggacgataccacggagaagaagcggttttgaaggagctaattataattatgac
A T G T I P R R R S G F E G A N Y N Y D 38
243 gttttggttaaagatactacacagctggaagacgagaacaaagaaattgatgaaagaagg
V L V K D T T Q L E D E N K E I D E R R 58
303 tctggacgaagaaatccctccttaaattcaggacttattttcgggaagagattcgaagaa
S G R R N P S L N S G L I F G K R F E E 78
363 gcagcggaggatttttaaatgacgacgaatctaggcaaattaatttggtttcccggggg
A A E D F L N D D E S R Q I N L V S R G 98
423 cgtagtaggctaccctttcacagcggacttatgcaaggcaaaagaaatccactacaagat
R S R L P F H S G L M Q G K R N P L Q D 118
483 aaccttagtgtcaagaggtcaagaccacagtttcatactggatttatgatgggtaaacgg
N L S V K R S R P Q F H T G F M M G K R 138
543 tttacgccggaagcagacgacttcgatctagaagagttcaagagaaaagcaggtcaaagg
F T P E A D D F D L E E F K R K A G Q R 158
603 ttacgttttccgacggaatgctatttggaaaataaccctccctctacggacagaacaat
L R F S D G M L F G K * 169
663 ccaataaagcctatttggtttcgttttcgttggcttacgtatgagattgtttgataata
723 gtactgatgtacagtaagcaacaaaaagatctaaagaactattttgtgaatctctttga
783 ttgctgtcaaggtcaatgaagacattgactagaataactagttatttatagaatgaccgt
843 aaattaattttatttgtttctcatatgtatcaaatacaaaccagaatgtctgttgttgcg
903 tgtgtatgacttaattgcttcttcgaatgtcagagaacagctgcaaactaccacataatg
963 taagtgtgtcctatatctggtgatgtaaacatcggaggtatacaagtttcgatattaaca
1023 acgaaacaattgaaatagtcaaatagttacaagtgtactagaaaatacgataagttaatt
1083 taaatcatcaggtcgactgctgcatagtttattactggaacggtcaaattattgttgtgg
1143 ataactcaaccgcgatcattgtatagcatcggcttagcccattgtttgtttatattaatt
1203 tattggaaaaggcgatttgtatccacgatgggggaaattatttatattgttttttacaat
1263 tgactcaaataatattatttgaccagtatttactttccgtgttcatagttcggtggtaca
1323 cctgtttgctttttttggtttggtgtaaatggtttcgtattgagggcatgaagcattttat
1383 ttccagatgtattcgtattcatcacaacgtaacatttcaaatgataggacattaggtata
1443 ctttgatattatatgtattatgttataaatagatatcagtta**gtcattagtctgtcttg**
1503 **ttg**tggaaaagtaaacgagagtgaatgtttcctaacctaaaaccttgagtagaagtgggt
1563 aaatcccggtataaatccctaattattatgtcaaagagagtaaaaagtttgtctgagaag
1623 aaaaagtggaaagagtatatgtgaaatactatttttcagttattttttttggccaaaaag
1683 ataaatgccattttatgaaataaaaaaaactaatgcggccgatcttcggagacaagcttt
1743 tattctatctttgcgattatcaacattactgtgtacgttagaatggaaattaatgaatat
1803 gcgtcttctcgagaccttgctggatttatttgcattttcattgcactttaaacatgaccc
1863 aaacatgtttcttctgaacaatcgttcaaacaaacaaacaaacgaacattggaatcgttg
1923 tggactagaggactgtatatgcgggaacttatttacatattcgtctacgatgtcctatgg
1983 tatatttcacagttgctgggcgtccggttatgtacatgtcttttaaatattaacaagatc
2043 acgtgacacctgctcatgcaacaacatgaacaagcgattttttggacaaaatatgtttgtt
2103 cattttagttgattgttaactttaatttgagactacgtttagtttcttgcatttttaatg
2163 tgtttgccacttgtaatgatccagacaaattgcagtggaaagtgttcactttgatcagcc
2223 atatctaagagttagcgacttgttcaaagaatgacacgagtgatctgttttgttcttaag
2283 gggtcctgatacacttaaatcgaccccaacagaagtaaaaaccgtttagatatcattata
2343 tttcacgaatttcactgtagcagcgttacaccttacggtgacggaaattatcaccctaat
2403 atttgaagttatggggtatg

Figure S3. ***Ophionotus victoriae*** **L-type SALMFamide precursor.** The DNA sequence of an assembled transcript (contig 2059646; lowercase, 2422 bases) encoding an L-type SALMFamide precursor protein (uppercase, 169 amino acid residues) is shown. cDNA sequencing was employed to confirm the section of the DNA sequence bounded by the PCR primer sequences (bold, underline). Bases in the sequenced cDNA that differ from the assembled transcript sequence are shown in underlined italics. The predicted signal peptide of the precursor protein is shown in blue and the four putative neuropeptides derived from this precursor are shown in red with C-terminal glycine residues that likely substrates for amidation shown in orange. Putative monobasic or dibasic cleavage sites (KR, RR, K) are shown in green and the asterisk shows the position of the stop codon. This sequence has been deposited in the GenBank database under accession number KM979353.
