# Supplementary material for: Reconstructing SALMFamide Neuropeptide Precursor Evolution in the Phylum Echinodermata: Ophiuroid and Crinoid Sequence Data Provide New Insights
Source: Front Endocrinol (Lausanne). 2015 Feb 2;6:2. doi: 10.3389/fendo.2015.00002 (PMC4313774; doi:10.3389/fendo.2015.00002)
Supplement: Supplementary file 1 [file Presentation_1.ZIP › Figure S4.PDF]

1 cc
3 ataatgcacgcctcccacgcccaacttaagtcagccaccgtggctgctctgaagttatga
63 aaaggtagcgcttgattggaatttctaacataagtcgtctgctcgtctgcgtggtggtgt
123 tttatagtattgactcttggcgc**gaagtggttgctaatacc**aaagacgccgtgcatggtt
183 ttggacttacgagggagcaggattgttttgcctttgtttgggataatttggtggtgcacc
243 tttgtta*a*ttcataaacaggtggcacacaagtttaagacttttaaactcagtgaggacta
303 taggatcttaagcagattctctgctccgttaagaacttaataaataagatggcccgagtg
M A R V 4
363 cggaatatatt*t*atactcttagcagcgatatgctgtcatgcaacgttatcgcacgcagaa
R N I F I L L A A I C C H A T L S H A E 24
423 gatgaagataccgaagaattgaaccacgaacaactagttgaatttgccaacaaaatcatg
D E D T E E L N H E Q L V E F A N K I M 44
483 ggtcaaatgaagttactagagtatgaattaggcattcaagaacataacgatggacaatta
G Q M K L L E Y E L G I Q E H N D G Q L 64
543 gatatggtgaaatccttaagtaagcgacaggctgtacgaccaggggggcggagctcctatg
D M V K S L S K R Q A V R P G G G A P M 84
603 aacgtacctgtgaaaatgtcagggttctcgtttggtaaacgcgatgctcaactggtcaga
N V P V K M S G F S F G K R D A Q L V R 104
663 aggtcagcaggtgccactccttcaaaattggcgggctttgcatttggaaaacgcggacaa
R S A G A T P S K L A G F A F G K R G Q 124
723 cctgttaaacgatcgtctgataacgaagcggaagaggaacaagagaagcgaggtgcaatg
P V K R S S D N E A E E E Q E K R G A M 144
783 gatgcatttgcttttggaaaacgtccatcaggtgaccccatgtctgcgttcagctttgga
D A F A F G K R P S G D P M S A F S F G 164
843 aaaaggcgtaatcccatgaactccttaagtgccctggcatttggtaagcgtgcaggtatg
K R R N P M N S L S A L A F G K R A G M 184
903 gatcccaattcgcttaatgctttcagttttggaaagcgcagggatccactcagcgcgttc
D P N S L N A F S F G K R R D P L S A F 204
963 agttttggaaaacgcggcatggattctctcagcgcgttcaatttcggcaaacggggtcgt
S F G K R G M D S L S A F N F G K R G R 224
1023 gatcatctgagcgctttcagttttggtaaacgtggacgaaatccaatgaatggtctcagt
D H L S A F S F G K R G R N P M N G L S 244
1083 gcatttgactttggaaaacgtggcgg*c*atggacgcatttgcatttggcaaacgcgagcag
A F D F G K R G G M D A F A F G K R E Q 264
1143 gaatataacgaggaaggtgcctttgacgacgaggctgaaaaacgcggttatgaaaatggc
E Y N E E G A F D D E A E K R G Y E N G 284
1203 c*tgtcgggatacgcatttggcaaaagagacactacagat*gatcagttaaatcacaatgac
L S G Y A F G K R D T T D D Q L N H N D 304
1263 gatacactgagaaccgattaacataaaactattcattcttatgccgcatgacatgaaatc
D T L R T D * 310
1323 atgaaaaagtgac**gtacggaaggactaaagt**gacattaaatccgaactattgaaaataac
1383 caatacacaatgaaaaattctacataatacatggagctttcgatcccattgtttgtttaa
1423 aaacaaatattgttaataaaaaggttactagtagttgctttcaaatgcctgccagtggac
1483 aatgtgttaagtgtgttcgataatgtttggatatctattaaaagtagtgtaatgttaacg
1543 taatatggcaaacgacaagatttcaacttcttgttaattcaaggtatgaactattggcaa
1603 aacgttaccaaaaggagcgatgaaggtcaagattgatagcaatcttcaaaacacatatga
1663 tataatgtagatctgtaataaaggtgtggtgtaatttactttttgagcccaattaatta
1723 aattgtataatgtattcgatcaagaaacacaaagttgtcagtagtacgggtcccagtaag
1783 ggtcctggtaactgtgtgatatgcgtattgtggatataaatggagaaatctgatgtgtgt
1843 attcatgcgtccattttaaataaatgcaatttcacaagctacttctagtttcagtcata
1903 gtgtctattgcgggaatacagtatgcatgcaataagggataagacggtattttacaattt
1963 atgtccattctaaattctgaattccacactttattaatactattacattgacaaagttat
2023 tagcatggtgtaaatggtgaatcaaacaacatcttcttgaagtaacatcgtgatatgtta
2083 cagccccacaagtaatcatagcgactaccaattgagtagttaatctttgacattgcaccg
2143 gaagtaaaacccttaccagaaaaaataccagcttttcccatagttttaaatatcccgtgt
2203 tggacaaatttgttgacttctataccacaaaatgcaccaattggcttatgtctaagaaga
2263 ctattaaaagtatcgctcttctgtcggcgatttagagtatagcctggtatttaaaaatcg
2323 atgtac

Figure S4. *Ophionotus victoriae* F-type SALMFamide precursor. The DNA sequence of a transcript (lowercase, 2328 bases) encoding an F-type SALMFamide precursor protein (uppercase, 310 amino acid residues). This was partially obtained from an assembled scaffold (64804) and then cDNA sequencing was employed to confirm the section of the DNA sequence bounded by the PCR primer sequences (bold, underline). Bases in the sequenced cDNA that differ from the assembled transcript sequence are shown in underlined italics. The predicted signal peptide of the precursor protein is shown in blue and the twelve putative neuropeptides derived from this precursor are shown in red, with C-terminal glycine residues that likely substrates for amidation shown in orange. Putative dibasic cleavage sites (KR, RR) are shown in green and the asterisk shows the position of the stop codon. This sequence has been deposited in the Genbank database under accession number KM979352.
