# Supplementary material for: Reconstructing SALMFamide Neuropeptide Precursor Evolution in the Phylum Echinodermata: Ophiuroid and Crinoid Sequence Data Provide New Insights
Source: Front Endocrinol (Lausanne). 2015 Feb 2;6:2. doi: 10.3389/fendo.2015.00002 (PMC4313774; doi:10.3389/fendo.2015.00002)
Supplement: Supplementary file 1 [file Presentation_1.ZIP › Figure S5.PDF]

```
1                                                            ga
3    aaactatacgttagaaggcatatgatagtcgtcaagtattcaagaattaagtcaccgatt
63   cgatacacttagatgatcgacgttgcaattactactttacaagccacgtctagaaagtga
123  taaatactgacgatacaacgggatagagagcctcgcaagaaacacacttccttctaaaaa
183  aaaccggtgacaataatatcagctcgtctaatagcgttagaattagctatacagatgttc
                                                           M  F    2
243  agtcagccccccttgtacctcctgttgacatggttccttttccagcactcgttattggca
      S  Q  P  P  L  Y  L  L  L  T  W  F  L  F  Q  H  S  L  L  A   22
303  cagggtcatacaggtgacaacattcgagagggaggtgttcgttataatagaccacatggc
      Q  G  H  T  G  D  N  I  R  E  G  G  V  R  Y  N  R  P  H  G   42
363  ggaggtgttccatcaaagaaggctaatactagttcagaaccaataaacaactggattaga
      G  G  V  P  S  K  K  A  N  T  S  S  E  P  I  N  N  W  I  R   62
423  gcattaccagtcttacatcgaggtctatactttggaaaaagagttccagcgaatggttac
      A  L  P  V  L  H  R  G  L  Y  F  G  K  R  V  P  A  N  G  Y   82
483  caacttgaagatcaatttcgtgatcctgcagttgcacatttggcatcaaaaagaaatcca
      Q  L  E  D  Q  F  R  D  P  A  V  A  H  L  A  S  K  R  N  P  102
543  gctttaagtgagtttatgttgggtaagagagatccaagctttagtagttacatgctcgga
      A  L  S  E  F  M  L  G  K  R  D  P  S  F  S  S  Y  M  L  G  122
603  aagagaaatcctcgattaagtgacctgatgttaggaaaacgagatcccagactgagtgat
      K  R  N  P  R  L  S  D  L  M  L  G  K  R  D  P  R  L  S  D  142
663  cttatgcttggaaagagagatccgcgactgagcgatctcatgctaggaaagagagatccg
      L  M  L  G  K  R  D  P  R  L  S  D  L  M  L  G  K  R  D  P  162
723  cgactgagcgaccttatgctaggaaagagagatccaggctttagtgactttacgtttggt
      R  L  S  D  L  M  L  G  K  R  D  P  G  F  S  D  F  T  F  G  182
783  aaaagagatgcactaggcgattttatgatgggcaaaagagaagcacgcttatcggattat
      K  R  D  A  L  G  D  F  M  M  G  K  R  E  A  R  L  S  D  Y  202
843  ataatgggaaaacgagatccacgtataagtgatttcattatgggccgaagagaattggga
      I  M  G  K  R  D  P  R  I  S  D  F  I  M  G  R  R  E  L  G  222
903  gaaaatgacgttcaacgacatatgggaaacaattactatgataacaaagtagaacatgaa
      E  N  D  V  Q  R  H  M  G  N  N  Y  Y  D  N  K  V  E  H  E  242
963  ggtaaacattatgttctaagtgatggaaaccgagaaaggatagaagacaacatgaataat
      G  K  H  Y  V  L  S  D  G  N  R  E  R  I  E  D  N  M  N  N  262
1023 gtgatttacgatgacactgatataccaaatcaagctgaagtttcggagttgcaagaattg
      V  I  Y  D  D  T  D  I  P  N  Q  A  E  V  S  E  L  Q  E  L  282
1083 gaatctagttcaagtgtaaagagaaaagctaagtttcagcgtccagtatatcctggaaac
      E  S  S  S  S  V  K  R  K  A  K  F  Q  R  P  V  Y  P  G  N  302
1143 ggtaaaacaccttctaacatttgggataacttcggagcaggcaagaggatgtcatctgta
      G  K  T  P  S  N  I  W  D  N  F  G  A  G  K  R  M  S  S  V  322
1203 ccggattatgaagatgaagaagaaaacgtgcaaaccgaaaccaaacgttcagcggatcca
      P  D  Y  E  D  E  E  E  N  V  Q  T  E  T  K  R  S  A  D  P  342
1263 aaaacatcagtgagaaggttcccgccagcagctcttcataaaggtctgtattttggaaaa
      K  T  S  V  R  R  F  P  P  A  A  L  H  K  G  L  Y  F  G  K  362
1323 agagcagcgacttgggcagacatgtaacatcacttcttaaatttagacaagttccgagtg
      R  A  A  T  W  A  D  M  *                                    370
1383 tcctaacgaaaaacgtgccttgctttagatcactccacttcggtacaagatcagaggagc
1443 ctcaacaaaattggagctataatgtttagtgatgaaatcaacaacttagatagacttatt
1503 tttcttaaagtcggtagaagtatggttttcataccgccaaatggaaattgtgaacacctt
1563 gttcactgatttatttttacaatattaatatatgattattgtattttagaacaaaatag
1623 tatcaattatttttatcttgaaaaattgtcagatatttcaaactcatttaatgataagt
1683 a
```

Figure S5. ***Antedon mediterranea*** **SALMFamide precursor.** The DNA sequence of a transcript (lowercase, 1683 bases) encoding a SALMFamide precursor protein (uppercase, 370 amino acid residues). The sequence data was first determined by manual assembly of three overlapping contig sequences (1781194, 446850 and 1694464) and then cDNA sequencing was employed to confirm the section of the DNA sequence bounded by the PCR primer sequences (bold, underline). Bases in the sequenced cDNA that differ from the assembled transcript sequence are shown in underlined italics. The predicted signal peptide of the precursor protein is shown in blue and the twelve putative neuropeptides derived from this precursor are shown in red, with C-terminal glycine residues that likely substrates for amidation shown in orange. Putative dibasic cleavage sites (KR, KK, RR) are shown in green and the asterisk shows the position of the stop codon. This sequence has been deposited in the Genbank database under accession number KM979351.
