# Supplementary material for: Reconstructing SALMFamide Neuropeptide Precursor Evolution in the Phylum Echinodermata: Ophiuroid and Crinoid Sequence Data Provide New Insights
Source: Front Endocrinol (Lausanne). 2015 Feb 2;6:2. doi: 10.3389/fendo.2015.00002 (PMC4313774; doi:10.3389/fendo.2015.00002)
Supplement: Supplementary file 1 [file Presentation_1.ZIP › Figure S6.PDF]

A

BLAST Query = *S. purpuratus* L-type SALMFamide precursor (130 letters)
Database = *Lytechinus variegatus* genome (version 0.4)
Hit = Scaffold 410, bases 42214-104113

```
S. pur: 1     MQVQQITVFLVACTLSVLVVAYAQEDAETVLLNRLRDIAARAAAGELPDFFADV---DDY 57
              MQVQQ+TVFLVACTLSVLVVAYAQEDA+  L+  +RDIA R AAG++PD+  ++   DDY
L. var: 1     MQVQQVTVFLVACTLSVLVVAYAQEDAQRALMETIRDIAMRVAAGDIPDYNPNMPNDDDY 60

S. pur: 58    KRGGKKNMGSIHSHSGIHFGKRRDSESSERARNTKRMRLHPGLLFGKRAPVQKWDQWQAQ 117
              KRGG+KN G I +H GI FGKRRDSESSER RN+KRMR H GLL GKR+PVQ W+QWQ +
L. var: 61    KRGGRKNRGGIKNHLGIQFGKRRDSESSERQRNSKRMRFHTGLLIGKRSPVQAWNQWQPE 120

S. pur: 118   DTYNPDWELGQFN 130
              +TYNPDW+LGQF+
L. var: 121   ETYNPDWQLGQFD 133
```

B

BLAST Query = *S. purpuratus* F-type SALMFamide precursor (266 letters)
Database = *Lytechinus variegatus* genome (version 0.4)
Hit = Scaffold 43, bases 173129-175795

```
S. pur: 1     MLFTMRVLVALALCLCFIAPSPVLSFTMPEEKFVENKMADVGEEGTGQNNINSIAKSLIR 60
              ML TMR+LVALALCLCF APSPVLSFT+PEEKF+E+KM DVGEE TGQN+INSIAKSLIR
L. var: 1     MLLTMRLLVALALCLCFYAPSPVLSFTLPEEKFIESKMPDVGEESTGQNDINSIAKSLIR 60

S. pur: 61    EVFGAAEEREMEAENEAEDEAELSLSKRTTGSTRPQREIRARAQYAARRPPVTTRSKFTF 120
              EVFG A+EREM+AE+E E+  E+SLSKRTTGS RPQREIRARAQ+A RRPP+TTRSKFT+
L. var: 61    EVFGGADEREMDAEDEGEEAGEMSLSKRTTGSNRPQREIRARAQFAGRRPPITTRSKFTW 120

S. pur: 121   GKRSSPTPVISRPLAEQLLEELQRNAEMSDDWRESDKLALLNDAALYDSLVDSHQVQKDA 180
              GKRSSP+ +ISRP AEQLLEELQRNAEMSD+WR+SD+ ALLNDAALYD+L+D HQVQKDA
L. var: 121   GKRSSPS-MISRPTAEQLLEELQRNAEMSDEWRDSDRQALLNDAALYDNLMDKHQVQKDA 179

S. pur: 181   YSAFSFGKRGMSAFSFGKRAQPSFAFGKRGLMPSFAFGKRPHGGSAFVFGRRDWAPREQD 240
              +S+FSFGKRGMS FSFGKR+ PSFAFGKRGLM SFAFGKRPHGGSAF+FGRRDW PRE D
L. var: 180   FSSFSFGKRGMSPFSFGKRSMPSFAFGKRGLMSSFAFGKRPHGGSAFIFGRRDWEPRETD 239

S. pur: 240   FANAAEESGPYKRGDLAFAFGKREDQ 266
                    ESG +KRG++AF+FGKR DQ
L. var: 241   L-----ESGLFKRGNVAFSFGKRADQ 260
```

Figure S6 BLAST analysis of genome sequence data from the sea urchin *Lytechinus variegatus* (http://www.echinobase.org/Echinobase/Blast/LvBlast) identifies homologs of the *S. purpuratus* L-type (A) and F-type (B) SALMFamide precursors. Putative SALMFamide neuropeptides are shown in red, with C-terminal glycine residues that likely substrates for amidation shown in orange, and putative cleavage sites are shown in green. The sequences of some of the predicted neuropeptides vary between the two species, but the general characteristics of the precursors are the same in both species with respect to the number and type of SALMFamides that they contain.
