# Supplementary material for: Reconstructing SALMFamide Neuropeptide Precursor Evolution in the Phylum Echinodermata: Ophiuroid and Crinoid Sequence Data Provide New Insights
Source: Front Endocrinol (Lausanne). 2015 Feb 2;6:2. doi: 10.3389/fendo.2015.00002 (PMC4313774; doi:10.3389/fendo.2015.00002)
Supplement: Supplementary file 1 [file Presentation_1.ZIP › Figure S7.PDF]

A

BLAST Query = *A. japonicus* L-type SALMFamide precursor (176 letters)
Database = *Leptosynapta tenuis* transcriptome
Hit = comp37837_c0_seq1

```
A. jap: 1    MKAYQIIVPAVMCVLAAILARTEADGELRILNNRLFELTKELEERLREQQLEDADLILTE  60
             MK  Q+++ +V+C+    L+RT A+ +L  +  +L+++TKELE  L   ++ED D    E
L. ten: 1    MKLNQLVILSVVCIATFFLSRTSAESKLESIRQKLYDVTKELEAELEGMEIED-DSGWYE  59

A. jap: 61   DGDQEIGMKKVVSRAWSPLVGQTGIAFGKR--TDGLDRARSQTDQRAKKTRSRSMFGNTA  118
             D D          R +   +   G++FGKR      D  R+  DQ   K   R +  ++A
L. ten: 60   DNDD------FSKRNYKTFIKNNGLSFGKRGAYSQEDNTRAIRDQSNAKN-PRGISRHSA  112

A. jap: 119  LPFGKRAG-----YIPHAQEIWDLQDAANNLDTFEEVPVKKRMGFTGNTGILLGKRNADD  173
             L FGKR        +P     +DLQ        +EE   K++   TG+T ++LGKR+ D
L. ten: 113  LTFGKRGADWEGVLVPG----YDLQ--------YEEPTRKRKYRLTGHTAMILGKRDVDQ  160

A. jap: 174  AQE  176
               E
L. ten: 161  ETE  163
```

B

BLAST Query = *A. japonicus* L-type SALMFamide precursor (176 letters)
Database = *Leptosynapta tenuis* transcriptome
Hit = comp38412_c0_seq1

```
A. jap: 1    MKAYQIIVPAVMCVLAAILARTEADGELRILNNRLFELTKELEERLREQQLEDADLILTE  60
             MK  Q+++ + +C+    L+RT A+ +L  +  +L+E+TKELEE L   +++D   +  E
L. ten: 1    MKLNQLVLLSAICIATFFLSRTSAESKLESIRQKLYEITKELEEELEGMEIDDDSQLYEE  60

A. jap: 61   DGDQEIGMKKVVSRAWSPLVGQTGIAFGKRTDGLDRARSQTDQRAKKTR-SRSMFGNTAL  119
             + D          R    LV   GI FGKR  G  R   + D+     R SR  +G+TAL
L. ten: 61   NDD-------FKRRGLKTLVRNNGITFGKR--GWLRPNDKDDRDQSNIRNSRGWYGHTAL  111

A. jap: 120  PFGKRAGYIPHAQEIWDLQDAANNLDTFEEVPVKKRMGFTGNTGILLGKRNADDAQE  176
              FGKR+ +  +           NN++     P+ KR   + +T +L GKR+A
L. ten: 112  SFGKRSPFPGY---------DVNNME-----PMTKRKLLSSHTAMLFGKRDAVQEN  153
```

C

```
37837  1    MKLNQLVILSVVCIATFFLSRTSAESKLESIRQKLYDVTKELEAELEGMEIEDDSGWYED  60
            MKLNQLV+LS +CIATFFLSRTSAESKLESIRQKLY++TKELE ELEGMEI+DDS  YE+
38412  1    MKLNQLVLLSAICIATFFLSRTSAESKLESIRQKLYEITKELEEELEGMEIDDDSQLYEE  60

37837  61   NDDFSKRNYKTFIKNNGLSFGKRGAYSQEDNTRAIRDQSNAKNPRGISRHSALTFGKRGA  120
            NDDF +R  KT ++NNG++FGKRG     D  +  RDQSN +N RG   H+AL+FGKR
38412  61   NDDFKRRGLKTLVRNNGITFGKRGWLRPND--KDDRDQSNIRNSRGWYGHTALSFGKRSP  118

37837  121  DWEGVLVPGYDLQYEEPTRKRKYRLTGHTAMILGKRDVDQETE  163
                   PGYD+   EP  KRK  L+ HTAM+ GKRD  QE
38412  119  ------FPGYDVNNMEPMTKRKL-LSSHTAMLFGKRDAVQEN   153
```

D

BLAST Query = *A. japonicus* F-type SALMFamide precursor (290 letters)
Database = *Leptosynapta tenuis* transcriptome
Hit = comp38152_c0_seq1

```
A. jap:  1                                 MAPPSIFLLISVSFLAVLHPSHTETLDHT  29
                                           M   +  L++ +
L. ten:  1                                 MGASTRLLVVFL-----------------  12

A. jap:  30  GAIVKEIQMELPHDMETAQL----LLQGQELRDLADELALDYLNERSDD--DDTFSNIAK  83
               ++  +Q   P   + A+        G  L++ A  L LDYL ER+ +  +D F
L. ten:  13  ATVIYSVQCMRPDQDDIAEFEDDNTQAGSRLKNFAKSLVLDYL-ERTGEFPEDGFEKRNM  71

A. jap:  84  REPGRSSTTNNKD--TMYEPFVRRGVPPYVVKVTYGKRSDDKRFKSPFMFGKREDLNGLD  141
                 R    N+KD   +   F RR V P   K T+GKRSD+              +N ++
L. ten:  72  AGQARE-IRNDKDFSKLVNSFGRRNVAPSFSKWTFGKRSDN--------------MNDME  116

A. jap:  142 KRGYSPFMFGKREMAQPHLSEKRARYSPFTFGKR-----------------DGADDEDEN  184
             KR  + F FGKR +      +++ R S F+FGKR                 DGAD ED+
L. ten:  117 KRRLNSFYFGKRSLEDALDMDEKRRMSSFSFGKRGPLNAFTFGKRLNTEQQDGADMEDD-  175

A. jap:  185 LEEVKRGGYSALYFGKRVPELAESDGGQSKLYFGKRGHRGGQFSQFKFGKREDGALGMDA  244
                 KR  YSA  FGKR  ++   D   S   FGKR     + S F FGKR D +   +
L. ten:  176 ----KR--YSAFAFGKRGDDM---DKRYSSFTFGKRDM-NKRLSSFTFGKRGDASEPYNY  225

A. jap:  245 NEDDEMEQNFEKKDAITQNKRFKSSFYLGKRNYVAENEDMEDLQDV 290
               D+E   + +  + +
L. ten:  226 IPDNEALLDSDDENTLPKIVLK 247
```

Figure S7 BLAST analysis of transcriptome sequence data from the sea cucumber *Leptosynapta tenuis* (O'Hara et al., 2014) identifies homologs of the *A. japonicus* L-type (A, B) and F-type (D) SALMFamide precursors. The putative SALMFamide neuropeptides are shown in red, with C-terminal glycine residues that likely substrates for amidation shown in orange, and putative cleavage sites are shown in green. Two L-type SALMFamide precursors were identified in *L. tenuis* (A, B) and comparison of their sequences (C) reveals a high level of sequence similarity, indicating that they have arisen by gene duplication in this species or in an ancestor of this species. The overall structural organization of the two L-type SALMFamide precursors in *L. tenuis* is similar to the L-type SALMFamide precursor in *A. japonicus*, comprising three homologous L-type SALMFamide neuropeptides. The F-type SALMFamide precursors in *A. japonicus* and *L. tenuis* (D) both comprise eight putative neuropeptides and six of these are aligned. The sequences and positions of the other two peptides in each species indicate that they have arisen independently.
