# Supplementary material for: Reconstructing SALMFamide Neuropeptide Precursor Evolution in the Phylum Echinodermata: Ophiuroid and Crinoid Sequence Data Provide New Insights
Source: Front Endocrinol (Lausanne). 2015 Feb 2;6:2. doi: 10.3389/fendo.2015.00002 (PMC4313774; doi:10.3389/fendo.2015.00002)
Supplement: Supplementary file 1 [file Presentation_1.ZIP › Figure S8.PDF]

A

BLAST Query = *P. miniata* L-type SALMFamide precursor (212 letters)
Database = *Luidia senegalensis* transcriptome
Hit = comp20078_c0_seq1

```
P. min:   1   MKLYPLLAVAVLFVAGPFRIIEAYSPFGGYHRALLGNVWVRASDNRARP--ASTPEEEAN   58
              MKLYPLLAVAVLFVAGP  I EAYSPFGGY+RALLGNVWVRA D+RAR    ST E+EAN
L. sen:   1   MKLYPLLAVAVLFVAGPLGICEAYSPFGGYNRALLGNVWVRA-DSRARAGGGSTVEDEAN   59

P. min:   59  EQRMTGAKRPAGSPVFHSALTYGKRA-----------DEADTDAAVERRAFHSALPFGKR   107
              E+R+TGAKRPAGSPVFHSAL+YGKRA           +E D     ERRA+HS + FGKR
L. sen:   60  EERLTGAKRPAGSPVFHSALSYGKRAGGGGGEEELVAEEEDGADGFERRAYHSGITFGKR   119

P. min:   108 TA-MDRRGLHSALPFGKRDDEEAEQDALMERRGFNSALMFGKRIHTALPFGKRGYHSALP   166
              TA MDRRG HSA+PFGK    EAE+ A   RRG+NSALMFGKR+H+ALPFGKR +HS LP
L. sen:   120 TADMDRRGFHSAMPFGK---REAEEAAARARRGYNSALMFGKRVHSALPFGKRAFHSGLP   176

P. min:   167 FGKRSDEE----EGTA-MERRGYHTGLPFGKRDDGTDAAVSEILSQLRSED   212
              FGKR  +E    EG A  ERRG+H+GLPFGKR    D  VS+ILSQLRS++
L. sen:   177 FGKRQPDEDQAGEGAAPAERRGFHSGLPFGKRGQDADDVVSDILSQLRSQE   227
```

B

BLAST Query = *P. miniata* F-type SALMFamide precursor (258 letters)
Database = *Luidia senegalensis* transcriptome
Hit = comp20908_c0_seq1

```
P. min: 1     MLRLVALLGIASLLVCQSAGQDAANAEEQEDFYPAERTNGFDNLKGIRDSD-DQHLHSAT   59
              M+R V LL +A+LL CQ A  +   A+ + D  P      FD   G  D D D  LHSA+
L. sen: 1     MMRYVLLLVVANLLGCQVAFAEPNQADGEGDLSP------FDG--GRPDFDLDGLLHSAS   52

P. min: 60    KRDVSDRQREIDLAAQQPFYPYGRRTDVPGRPSGFVFGKRGQ-FFLPYQ--YQKRELDEV   116
              KR+ SDRQRE++LAAQQPF PYGRRTDVPG      FGKRGQ +F+P Q  + KR+L EV
L. sen: 53    KREGSDRQREVELAAQQPFNPYGRRTDVPGW---LTFGKRGQQYFVPAQDPFPKRDLAEV   109

P. min: 117   NPYSVAKRDDELTGLEEELDASKRSNGPYSMSGLRSLTFGKREDAWSPEKRADLFRSYAF   176
              NPYSV+KRD E+  L     D  +    PY+++GL SL+FGKR          DLFRSYAF
L. sen: 110   NPYSVSKRDAEMADLLA--DEKRGGPNPYALNGLGSLSFGKRG--------GDLFRSYAF   159

P. min: 177   GKRALGSNFAFGKRGYSSFDFGKRAGLGSSFTFGKRGVPDVDRFADEDAL-----LQDNK   231
              GKRALGS+FAFGKRG SSF FGKR G                   DE AL        +K
L. sen: 160   GKRALGSSFAFGKRGLSSFAFGKRGG------------------DDEGALPDDAAAAASK   201

P. min: 232   RALGSSFSFGKRSGLSSFTFGKRAGER   258
              RALGSSFSFGKR GLSSF FGKRAG+R
L. sen: 202   RALGSSFSFGKRGGLSSFAFGKRAGQR   228
```

Figure S8 BLAST analysis of transcriptome sequence data from the starfish *Luidia senegalensis* (O'Hara et al., 2014) identifies homologs of the *P. miniata* L-type (A) and F-type (B) SALMFamide precursors. Putative SALMFamide neuropeptides are shown in red, with C-terminal glycine residues that likely substrates for amidation shown in orange, and putative cleavage sites are shown in green. In both species the L-type SALMFamide precursors comprise seven neuropeptides. The *P. miniata* F-type SALMFamide precursor comprises nine putative neuropeptides and homologs of eight of these are present in the *L. senegalensis* F-type SALMFamide precursor; a homolog of the seventh putative neuropeptide in *P. miniata* precursor (AGLGSSFTFamide) is absent in *L. senegalensis*.
