# Supplementary material for: Reconstructing SALMFamide Neuropeptide Precursor Evolution in the Phylum Echinodermata: Ophiuroid and Crinoid Sequence Data Provide New Insights
Source: Front Endocrinol (Lausanne). 2015 Feb 2;6:2. doi: 10.3389/fendo.2015.00002 (PMC4313774; doi:10.3389/fendo.2015.00002)
Supplement: Supplementary file 1 [file Presentation_1.ZIP › Figure S9.PDF]

A

BLAST Query = *O. victoriae* L-type SALMFamide precursor (169 letters)
Database = *Ophiothrix angulata* transcriptome
Hit = *comp44302_c0_seq1*

```
O. vic: 1   MRLQPLLVFCICALVPFAATGTIPRRRSGFEGANYNYDVLVKDTTQLEDENKEIDERRSG  60
            MRLQ  LV  ICA++P  A G +PRR S  E   YNYD +VKD  Q+ +++KEI+ERRSG
O. ang: 1   MRLQARLVLFICAIIPVIAAGVVPRRASK-ESPTYNYDAMVKDP-QMANQDKEIEERRSG  58

O. vic: 61  RRNPSLNSGLIFGKRFEEAAEDFLNDD---ESRQINLVSRGRSRLPFHSGLMQGKRNPLQ  117
            R    LNSGL+FGKRFEEAA+DF+ND+   + +Q  +  RGRS+LPFHS LMQGKR+ L+
O. ang: 59  RSRTYLNSGLLFGKRFEEAADDFINDENTNQQQQFTVEVRGRSKLPFHSALMQGKRSSLE  118

O. vic: 118 DNLSVK-----RSRPQFHTGFMMGKRFTPE--ADDFD----LEEFKRKAGQRLRFSDGML  166
            D          + + +FH+  ++GKR+ PE   D  D    ++EFKRK G RLR+S+GM
O. ang: 119 DEFDSSFSKRGKQKAKFHSAMLLGKRY-PEFGIDAVDGVQNIDEFKRK-GSRLRWSNGMA  176

O. vic: 167 FGK  169
            FG+
O. ang: 177 FGR  179
```

B

BLAST Query = *O. victoriae* F-type SALMFamide precursor (310 letters)
Database = *Ophiothrix angulata* transcriptome
Hit = *comp56008_c0_seq1*

```
O. vic: 1   MARVRNILILLAAICCHATLSHAED---EDTEELNHEQLVEFANKIMGQMKLLEYELGIQ  57
            MARVR++++L AA+CC+ ++SHAE+   +  +EL H++LVE A++I  ++++LE  LGIQ
O. ang: 1   MARVRHVVLLFAALCCYTSISHAEENEYQTNQELEHDKLVELASRIAKEVRILELGLGIQ  60

O. vic: 58  EHNDG----QLDMVKSLSKRQAVRP---GGGAPMNVPVKMSGFSFGKRDAQLVRRSA---  107
            ++N+       D++KSL KRQA  P   G G PMNVPVKMSGF+FGKRD QLVRRSA
O. ang: 61  QYNEDDTEDNTDIIKSLQKRQAATPRSGGSGLPMNVPVKMSGFAFGKRDGQLVRRSAKSG  120

O. vic: 108 GATPSKLAGFAFGKRGQPVKRSSDNEAEEE-QEKRGAMDAFAFGKRPSGDPMSAFSFGKR  166
            G  P KLAGFAFGKRGQPVKRS+++E EE+ +EKR AMDAF FGKR S   +S FSF KR
O. ang: 121 GDKPVKLAGFAFGKRGQPVKRSTNDELEEDGEEKRAAMDAFTFGKRISDQELSPFSFEKR  180

O. vic: 167 RNPMNSLSALAFGKRAGMDPNSLNAFNFGKRR-DPLSAFSFG-KRGMDS--LSAFNFGKR  222
            R+P   LSAL FGKR GM P+S++AF+FGKRR DPLSAFSFG KR MD   LSAF+FGKR
O. ang: 181 RDP-TGLSALTFGKR-GMHPSSMSAFSFGKRRMDPLSAFSFGKKRAMDPAGLSAFSFGKR  238

O. vic: 223 GRD--HLSAFSFGKRGRNPMNGLSAFDFGKRGGMDAFAFGKREQEYNEEGAF---DDEAE  277
            G D   LSAFSFGKRG  P +GLSAF FGKR GM+AF FGKRE E  EE AF    ++ E
O. ang: 239 GMDPSALSAFSFGKRGTGP-SGLSAFSFGKRMGMNAFTFGKREGE-EEETAFKKNTNDDE  296

O. vic: 278 KRGYENGLSGYAFGKRDT--TDDQLNHNDDTLRTD  310
            KR   NGLS + FGKRDT   +++LN ND+TLR D
O. ang: 297 KRAGYNGLSQFTFGKRDTEAVEERLN-NDETLRDD  330
```

Figure S9 BLAST analysis of transcriptome sequence data from the brittle star *Ophiothrix angulata* (O'Hara et al., 2014) identifies homologs of the *O. victoriae* L-type (A) and F-type (B) SALMFamide precursors. Putative SALMFamide neuropeptides are shown in red, with C-terminal glycine residues that likely substrates for amidation shown in orange, and putative cleavage sites are shown in green. The sequences of some of the predicted neuropeptides vary between the two species, but the general characteristics of the precursors are the same in both species with respect to the number and type of SALMFamides that they contain.
